# Bioinformatic and expression analysis of the *Brassica napus* L. cyclophilins

**DOI:** 10.1038/s41598-017-01596-5

**Published:** 2017-05-04

**Authors:** Patrizia Hanhart, Melanie Thieß, Khalid Amari, Krzysztof Bajdzienko, Patrick Giavalisco, Manfred Heinlein, Julia Kehr

**Affiliations:** 1 Molecular Plant Genetics, Universität Hamburg, Biozentrum Klein Flottbek, Ohnhorststraße 18, 22609 Hamburg, Germany; 2 Université de Strasbourg, CNRS, IBMP UPR 2357, 12 rue du Général Zimmer, F-67000 Strasbourg, France; 3 Max-Planck-Institut für Molekulare Pflanzenphysiologie, Wissenschaftspark Potsdam-Golm, Am Mühlenberg 1, 14476 Potsdam, Germany

## Abstract

Cyclophilins (CYPs) are a group of ubiquitous proteins characterized by their ability to bind to the immunosuppressive drug cyclosporin A. The CYP family occurs in a wide range of organisms and contains a conserved peptidyl-prolyl *cis*/*trans* isomerase domain. In addition to fulfilling a basic role in protein folding, CYPs may also play diverse important roles, e.g. in protein degradation, mRNA processing, development, and stress responses. We performed a genome-wide database survey and identified a total of 94 *CYP* genes encoding 91 distinct proteins. Sequence alignment analysis of the putative BnCYP cyclophilin-like domains revealed highly conserved motifs. By using RNA-Seq, we could verify the presence of 77 *BnCYP* genes under control conditions. To identify phloem-specific BnCYP proteins in a complementary approach, we used LC-MS/MS to determine protein abundances in leaf and phloem extracts. We detected 26 BnCYPs in total with 12 being unique to phloem sap. Our analysis provides the basis for future studies concentrating on the functional characterization of individual members of this gene family in a plant of dual importance: as a crop and a model system for polyploidization and long-distance signalling.

## Introduction

As one of the most important crops for nutritional oil, fodder, biodiesel, chemical and pharmaceutical products, *Brassica napus* (oilseed rape or canola) is widespread in agriculture, especially in the European Union, China, and Canada. Beside its essential agricultural significance, *B. napus* has also become a model plant for studying long-distance signalling^1–5^ and evolutionary consequences of polyploidization^6, 7^. *Brassica napus* is used as a model plant for studying long-distance signalling, because methods for the collection of sufficient quantities of pure xylem and phloem sap are well established^2, 4^. Compared to crops like wheat, soybean or rice, the domestication of *B. napus* was more recent. It is assumed that chromosome doubling occurred after spontaneous hybridization between *Brassica rapa* (Asian cabbage or turnip rape, 2*n* = 2 × 10 = 20, genome AA) and *Brassica oleracea* (Mediterranean cabbage, 2*n* = 2 × 9 = 18, genome CC)^8^. The profitable cultivation of *B. napus* (genome AACC, 2*n* = 38) has high importance for the economy^9^.

A group of proteins involved in diverse fundamental cellular functions in many different organisms is called immunophilins, originally discovered as receptors for immunosuppressive drugs in mammals. The family of immunophilins consists of two major groups, FK506-binding proteins (FKBPs)^10, 11^ and cyclophilins (CYPs)^12, 13^. Despite the lack of sequence and structure similarity, FKBPs and CYPs each possess a conserved domain responsible for their common peptidyl-prolyl *cis/trans* isomerase (PPIase) activity, catalyzing the rate-limiting rotation of X-proline peptide bonds from a *cis* to a *trans* conformation^14^. These domains are called the FKBP and cyclophilin-like domain (CLD), respectively. An additional group of proteins exhibiting PPIase activity, the parvulins, cannot be classified as immunophilins in a strict sense, since they do not bind to any known immunosuppressant molecule^15^. The drugs of CYPs and FKBPs are cyclosporin A (CsA) and FK506/rapamycin, respectively, that bind to the catalytic pocket of the PPIase domain^16^, thereby inhibiting its activity and forming a high-affinity binding site for the interaction with calcineurin^17^. However, these drugs do not occur naturally in cells, therefore the consequences of drug treatment have clinical but no physiological relevance^18^.

Human CYPs were first characterized by their ability to bind the drug cyclosporin A in 1984^12^. The first plant CYPs were described in 1990, where CYP cDNA sequences were identified in tomato (*Lycopersicon esculentum*), maize (*Zea mays*), and oilseed rape (*B. napus*)^19^. The ubiquitous CYPs show highly conserved structural features^20^ and are involved in several fundamental cellular functions, including protein folding^21–23^, protein trafficking^24^, signalling^25–29^, pathogen response^30^, apoptosis^31^, RNA-binding, regulation of gene expression or transcription^32–36^, and plant stress responses^37–40^.

Interestingly, CYPs were found to be a prominent and abundant class of proteins in the phloem long-distance system of higher plants including castor bean and oilseed rape^2, 41^. Phloem sieve elements lose their ability for transcription and translation during their maturation into transport tubes^42^. However, phloem exudate contains a complex set of proteins, some of which have been implicated with long-distance signalling^43^. Such signalling proteins are imported from the adjacent companion cells through plasmodesmata. It was proposed that phloem CYPs might act as molecular chaperones in this process, potentially involved in refolding of non-cell-autonomous proteins after entry into the translocation stream^2, 41^.

The growing number of sequenced genomes allowed the identification of whole sets of CYPs in various organisms by sequence comparisons. 19 CYPs were detected in the human genome, whereas *Saccharomyces cerevisiae* possesses 8, *Schizosaccharomyces pombe* 9, *Caenorhabditis elegans* 17, *Drosophila melanogaster* 14^44, 45^, and the fungus *Leptosphaeria maculans* 12^46^ CYPs. Compared to other organisms, plants have a higher number of CYPs with 29 encoded in *A. thaliana* ^47–49^, and 27 in rice^37^. To date, soybean (*Glycine max*) is reported to have the largest set of CYPs with 62 members^50^.

Based on the recently sequenced *B. napus* genome^51^, the major aim of the present study was the identification and classification of CYP-like proteins in this economically important species. A total number of 94 genes belonging to the *CYP* gene family (resulting in 91 different CYP proteins) could be identified in the sequenced *B. napus* cultivar ‘Darmor-bzh’. By applying transcriptome analysis of the *B. napus* cultivar ‘Drakkar’ we could confirm the expression of 77 *BnCYP* genes in leaves. To identify CYP proteins specifically occurring in phloem sap, we performed complementary protein analyses by LC-MS/MS on leaf and phloem extracts and found 26 different BnCYP proteins in total, 12 only present in phloem sap.

## Results and Discussion

### Identification of cyclophilins in the *Brassica napus* genome

Putative CYPs of *B. napus* containing full length or partial CYP-like domains were identified by BLASTp of *A. thaliana* CYPs^47, 48^. The 94 *B. napus* (cultivar ‘Darmor-bzh’) gene sequences determined by this approach resulted in 91 distinct proteins which were subjected to additional analysis like sequence alignments with the respective Arabidopsis homologs and the verification of CLDs. The analysis showed that *B. napus* contains the largest *CYP* gene family known to date with 94 genes followed by soybean (*Glycine max*) with 62 members^50^.

As proposed by Romano *et al*.^47^ and He *et al*.^48^ we used the nomenclature BnCYP (*Brassica napus* cyclophilin) followed by the molecular weight and a consecutive number for genes encoding proteins with similar molecular weight. We used the molecular weight of the immature proteins as the basis, because the prediction of potential cleavable signal peptides by different prediction tools was not unambiguous. Table 1 summarizes the information about all identified BnCYPs.

**Table 1:** Nomenclature, gene name, accessions, molecular weight, protein sequence length, information about aligned amino acids, theoretical isoelectric point, and predicted exons of the *B. napus* CYP family.

Name	Gene Name	GenBank Accession	kDa	aa	CLD	pI (theoretical)	Exons
BnCYP5	BnaA01g25420D	CDY37322.1	5.7	49	2–42	8.7	1
BnCYP7-1	BnaA09g37110D	CDY06069.1	7.2	66	1–66	10.7	3
BnCYP7-2	BnaA01g36790D	CDY60385.1	7.3	64	1–61	4.5	1
BnCYP7-3	BnaA01g36800D	CDY60386.1	7.3	65	1–64	4.9	1
BnCYP8	BnaCnng42430D	CDY63671.1	8.2	71	9–65	9.1	2
BnCYP10-1	BnaC02g14160D	CDX96235.1	10.1	92	6–55	4.7	2
BnCYP10-2	BnaC06g10280D	CDY30664.1	10.5	94	1–68	6.7	2
BnCYP12-1	BnaC02g10590D	CDY02830.1	12.5	109	52–96	9.8	5
BnCYP12-2	BnaAnng41240D	CDY72459.1	12.8	117	43–116	9.0	3
BnCYP13	BnaA02g07550D	CDY32693.1	13.1	116	8–64	8.3	4
BnCYP14-1	BnaC04g41450D	CDX97794.1	14.0	127	1–123	5.6	1
BnCYP14-2	BnaC04g41430D	CDX97796.1	14.2	127	1–124	5.8	1
BnCYP14-3	BnaC04g41440D	CDX97795.1	14.2	127	1–123	6.0	1
BnCYP14-4	BnaC08g28870D	CDX71987.1	14.3	131	1–109	6.1	1
BnCYP14-5	BnaC09g34640D	CDX80278.1	14.4	133	84–132	9.4	3
BnCYP14-6	BnaA01g25470D	CDY37327.1	14.9	138	2–137	4.6	3
BnCYP16	BnaA02g10200D	CDY28828.1	16.1	148	3–124	4.4	3
BnCYP17-1	BnaA01g25460D	CDY37326.1	17.3	161	68–134	7.7	3
BnCYP18-1	BnaC04g09170D/BnaA05g08140D	CDX75068.1/CDX84155.1	18.2	164	7–163	8.9	5
BnCYP18-2	BnaC01g03590D	CDX69094.1	18.2	172	1–171	8.9	1
BnCYP18-3	BnaA01g02340D	CDX75468.1	18.2	172	1–172	8.9	1
BnCYP18-4	BnaA08g16920D/BnaC03g60160D	CDX90292.1/CDY12100.1	18.3	172	2–172	8.3	1
BnCYP18-5	BnaA09g08780D	CDY47469.1	18.4	171	1–171	8.3	1
BnCYP18-6	BnaA06g37360D	CDY22414.1	18.4	172	2–171	8.3	1
BnCYP18-7	BnaC07g47630D	CDX72741.1	18.4	172	2–171	8.3	1
BnCYP18-8	BnaC09g09060D	CDY26779.1	18.4	171	1–171	8.3	1
BnCYP19-1	BnaA09g35540D	CDY27248.1	19.6	182	1–174	7.7	1
BnCYP19-2	BnaC08g26990D	CDX72175.1	19.7	183	1–174	7.0	1
BnCYP20	BnaA01g36700D	CDY70394.1	20.9	192	15–191	8.2	1
BnCYP21-1	BnaCnng08980D	CDY38037.1	21.0	193	22–191	9.2	5
BnCYP21-2	BnaC04g06640D	CDX91486.1	21.6	200	23–200	8.2	7
BnCYP21-3	BnaAnng15590D	CDY58941.1	21.7	201	28–199	8.9	6
BnCYP21-4	BnaC03g12390D	CDX71155.1	21.7	201	26–199	9.3	7
BnCYP21-5	BnaC04g54560D	CDY55458.1	21.7	201	28–199	8.9	6
BnCYP21-6	BnaAnng17350D	CDY61026.1	21.8	201	26–199	9.3	7
BnCYP21-7	BnaA05g06380D	CDX74892.1	21.8	204	27–204	8.2	7
BnCYP21-8	BnaC04g45890D	CDX93307.1	21.9	205	28–205	8.2	6
BnCYP21-9	BnaC09g34020D/BnaA10g29520D	CDX80340.1/CDY52123.1	22.0	204	31–202	8.9	7
BnCYP22-1	BnaCnng32070D	CDY57430.1	22.0	191	133–176	6.5	4
BnCYP22-2	BnaA04g22160D	CDY18371.1	22.3	207	26–207	8.1	7
BnCYP22-3	BnaC04g41460D	CDX97793.1	23.0	215	55–212	5.7	2
BnCYP24-1	BnaC08g26840D	CDX72190.1	24.0	224	53–222	6.5	8
BnCYP24-2	BnaA09g35470D	CDY27255.1	24.0	224	53–222	6.5	8
BnCYP24-3	BnaA10g12350D	CDY33029.1	24.3	222	86–140	9.2	4
BnCYP24-4	BnaA08g10930D	CDX76307.1	24.4	223	42–215	6.5	7
BnCYP24-5	BnaC01g03530D	CDX69100.1	24.6	224	44–215	7.1	7
BnCYP24-6	BnaA01g02260D	CDX75476.1	24.6	224	43–215	7.1	7
BnCYP24-7	BnaC03g65620D	CDY17501.1	24.6	225	44–217	6.2	7
BnCYP25-1	BnaA08g20010D	CDY46429.1	25.4	226	26–198	8.4	8
BnCYP25-2	BnaC08g48540D	CDY52765.1	25.5	226	23–198	8.4	8
BnCYP25-3	BnaA09g29400D	CDY16206.1	25.6	226	20–200	6.5	8
BnCYP25-4	BnaA03g21710D	CDX83335.1	25.9	231	79–230	8.9	6
BnCYP25-5	BnaC03g25990D	CDX95718.1	25.9	231	79–230	8.9	6
BnCYP26-1	BnaA05g30740D	CDY24675.1	26.3	234	74–230	8.7	7
BnCYP26-2	BnaA08g08200D	CDX76580.1	26.3	239	151–213	6.5	4
BnCYP26-3	BnaC05g45190D	CDY05284.1	26.3	234	74–230	9.0	7
BnCYP26-4	BnaA04g17840D	CDY29643.1	26.3	248	77–245	6.1	1
BnCYP26-5	BnaCnng32180D	CDY57615.1	26.5	236	76–232	8.7	7
BnCYP27-1	BnaC05g44950D	CDY05308.1	27.1	247	6–200	4.9	3
BnCYP27-2	BnaC08g31970D	CDX76611.1	27.4	253	81–249	8.7	7
BnCYP27-3	BnaC03g05790D	CDX70495.1	27.8	257	80–252	9.6	7
BnCYP27-4	BnaA09g39610D	CDY11395.1	27.9	258	86–254	9.0	7
BnCYP27-5	BnaA03g04200D	CDX78508.1	27.9	256	79–251	9.7	7
BnCYP28-1	BnaA04g27460D	CDY58761.1	28.1	258	86–254	8.8	7
BnCYP28-2	BnaC04g21530D	CDX93994.1	28.6	261	89–257	8.9	7
BnCYP30-1	BnaC08g08050D	CDY12400.1	30.4	280	68–267	6.3	2
BnCYP30-2	BnaA08g07170D	CDY41762.1	30.5	281	75–270	5.9	2
BnCYP31	BnaC07g00280D	CDY05775.1	32.0	277	59-173	4.8	7
BnCYP34-1	BnaC02g22620D	CDY45858.1	34.5	320	92–314	9.1	3
BnCYP34-2	BnaA02g16680D	CDY49138.1	34.5	320	91–314	9.1	3
BnCYP37-1	BnaC01g21230D	CDY35601.1	37.4	340	161–319	6.1	5
BnCYP37-2	BnaA01g17950D	CDY42959.1	37.5	340	161–316	6.6	5
BnCYP40-1	BnaC07g05260D	CDX71247.1	40.3	361	3–175	6.3	8
BnCYP40-2	BnaA07g04040D	CDY36653.1	40.3	361	3–175	6.0	8
BnCYP47-1	BnaC08g10870D	CDY43466.1	47.1	418	157–312	8.2	7
BnCYP47-2	BnaA05g33830D	CDY37518.1	47.2	433	254–433	5.1	7
BnCYP47-3	BnaC05g48850D	CDY49057.1	47.4	436	257–436	5.0	6
BnCYP49	BnaA05g24020D	CDX97677.1	49.8	459	273–438	6.6	12
BnCYP50	BnaC05g38110D	CDX98564.1	51.0	470	284–445	5.7	12
BnCYP52	BnaC01g05170D	CDX68936.1	52.4	467	6–186	8.6	11
BnCYP55	BnaA01g03810D	CDX75321.1	55.3	492	6–185	7.7	10
BnCYP62	BnaAnng12550D	CDY53714.1	62.0	556	4–177	10.7	13
BnCYP65-1	BnaA06g24990D	CDY08725.1	65.0	597	335–508	8.0	11
BnCYP65-2	BnaC03g48580D	CDY36499.1	65.1	597	335–508	7.3	11
BnCYP67	BnaC04g20680D	CDX93909.1	67.3	612	77–251	10.6	15
BnCYP70-1	BnaC03g54740D	CDY08708.1	70.1	622	465–619	6.0	13
BnCYP70-2	BnaA06g18830D	CDX99192.1	70.2	622	465–619	6.0	13
BnCYP86-1	BnaA01g04590D	CDX75243.1	86.9	765	3–174	11.8	13
BnCYP86-2	BnaC01g06080D	CDX68845.1	87.0	765	3–174	11.7	13
BnCYP112	BnaC03g71020D	CDY10512.1	112.6	992	1–176	5.9	18
BnCYP146	BnaA03g29520D	CDX74095.1	146.1	1268	1108–1264	6.0	13

### The cyclophilin-like domain

Since CYPs are characterized by the highly conserved CLD, its occurrence in the identified potential CYP sequences was verified. Most of the BnCYPs contained full length CLDs, whereas CYPs with a molecular weight below 17 kDa have partial CLDs missing essential residues or whole secondary structure parts and thereby might be lacking PPIase activity.

As a consequence of the CLD analysis, two proteins annotated as CYPs in the first place, BnaC05g10020D (30 kDa) and BnaC04g45810D (54 kDa), were excluded from subsequent studies. In addition, for one of the putative low molecular weight CYPs, BnCYP7-1, no CLD was predicted. Nevertheless, it was retained, because a BLASTp search and sequence alignment suggested a partial CLD.

**Figure 1:**
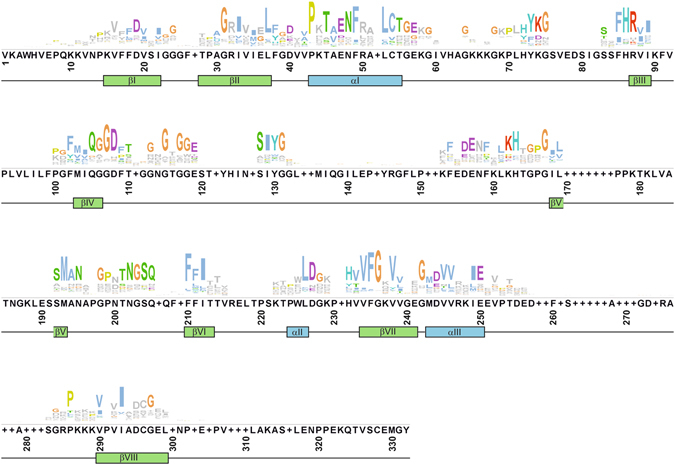
Conserved sequence of the *B. napus* cyclophilin-like domain. CYP protein sequences were cropped to the predicted sequence for the CLD and aligned with the secondary structure of hCypA. The conserved sequence of 91 predicted CLDs highlights structural features and functional residues. Gaps represent insertions in very few members of this protein family.

Figure 1 depicts the conserved sequence of all identified *B. napus* CLDs aligned with the secondary structure of human cyclophilin A (hCYPA) as a reference (for a detailed view of the multiple alignment see Supplementary Fig. S1). The crystal structure of hCYPA shows eight β-strands forming a β-barrel structure, plus two additional α-helices located at the top and bottom^52^. Human CYPA is often referred to as the “archetypal” CYP. Residues important for CYP function as well as residues promoting the secondary structure are conserved, whereas gaps in the conserved sequence represent insertions in individual members of this protein family. In previous studies it has been demonstrated that the highly conserved amino acid W121 of hCYPA (Fig. 1, W225) is required for CsA binding and interacts directly with the phosphatase calcineurin, but it is not essential for PPIase activity^53, 54^. This amino acid was not present in 57 out of the 91 BnCYP proteins. Further highly conserved amino acids fundamental for the PPIase activity of hCYPA are R55 (Fig. 1, R87), F60 (Fig. 1, F102;), and H126 (Fig. 1, H232)^53^. Three single mutations of these amino acids reduced the original activity of the human wild-type isomerase to less than 1%. However, the mutant proteins were still able to bind CsA. Since some of the BnCYPs did not possess all of these three amino acids, their PPIase activity needs to be experimentally confirmed. The motif VXGXV of the hydrophobic region around β-sheet-VII is reported to be conserved among all AtCYP proteins^47^. This motif was also present in 62 BnCYP protein sequences (Fig. 1, V234, G236, V238).

Exceptional insertions of amino acids in plant CYPs between β-sheet-I and β-sheet-II (β-I/β-II), β-sheet-IV and β-sheet-V (β-IV/β-V), and β-sheet-VI and α-helix-II (β-VI/α-II)^20^ have also been observed in this study. In more detail, 3 amino acids are inserted between β-I/β-II (Fig. 1, residues 25–27) in 16 BnCYPs. Yet, 45 BnCYPs do not show this insertion. Interestingly, we also observed deletions in 30 BnCYPs in this region. Only a minority of BnCYPs contains an insertion between β-IV/β-V (Fig. 1, residues 133–152) with 1 to 19 amino acids in length, but also here deletions occurred in some cases. Insertions between β-VI/α-II (Fig. 1, residues 216–221) are only present in 5 BnCYPs with medium and higher molecular masses. An additional insertion between α-helix-I and β-sheet-III (α-I/β-III junction) has been described by Romano *et al*.^47^. Here, 8 to 11 amino acids are inserted in several AtCYPs and 3 to 4 amino acids in chloroplast variants. For *B. napus* a 7 amino acid insertion occurs in several CYPs. For example, except for BnCYP18-1, all 18 and 19 kDa BnCYPs show this insertion.

### Domain architecture of cyclophilins and homology

Depending on the domains present, CYPs are classified as single- (SD) and multi-domain (MD) forms^48^. SD CYPs contain only a CLD which was the case for most of the BnCYPs with a rather low molecular weight (18–30 kDa). While there are mostly two BnCYP homologs with high sequence identity (>80%) corresponding to each AtCYP, caused by polyploidization, there are only a few exceptions with only one corresponding BnCYP homolog. One example is BnCYP18-1 which is the only *B. napus* homolog of AtCYP18-2 with a full length CLD (with 95% sequence identity, Table 2). It has been shown that the Arabidopsis CYP AtCYP18-2 is recruited by AtSKIP to the nucleus to regulate pre-mRNA splicing^55^. Because of the high homology, the oilseed rape variant might fulfil a similar role in the nucleus of *B. napus* cells.

**Table 2:** The Arabidopsis cyclophilin family, localization, functions and corresponding *Brassica* homologs.

AtCYP	Gene	Subcellular Localization*	Comments	BnCYP homolog (Identity)
AtCYP18-1	At1g01940	Cytosol (p^47, 48^)	up-regulated during heat stress^81^	BnCYP21-1 (98%)
AtCYP18-2	At2g36130	Cytosol (p^47, 48^)	pre-mRNA splicing, down-regulated after pathogen treatment^48, 55^	BnCYP18-1 (95%), BnCYP13 (79%), BnCYP8 (56%)
AtCYP18-3	At4g38740	Cytosol (p^47, 48^)	plant growth (stem elongation, shoot branching), hormone signalling (links light signal receptors to brassinosteroids), pathogen defence/ETI (by binding of pathogenic proteins/RNA (inhibition of replication (TBSV), interacts with *A. tumefaciens* VirD2 protein, *P. syringae* AvRpt2 protease and the plant RIN4 protein)), up-regulated after salt treatment^48, 82–89^	BnCYP18-4 (93%), BnCYP18-6 (92%), BnCYP18-7 (92%)
AtCYP18-4	At4g34870	Cytosol (p^47, 48^)	pathogen infection (interaction with *A. tumefaciens* VirD2 protein), down-regulated after salt and cytokinin treatment^48, 85^	BnCYP18-2 (83%), BnCYP18-3 (83%), BnCYP17-1 (74%), BnCYP10-1 (35%)
AtCYP19-1	At2g16600	Cytosol (p^47, 48^)	seed development, pathogen defence (ROS production, inhibiting pathogen growth), up-regulated after *P. syringae* infection, down-regulated after ABA treatment^30, 48, 90^	BnCYP18-5 (92%), BnCYP18-8 (92%), BnCYP26-4 (66%), BnCYP22-3 (65%), BnCYP20 (62%), BnCYP14-1 (62%), BnCYP14-2 (62%), BnCYP14-3 (60%), BnCYP14-6 (52%), BnCYP16 (50%), BnCYP7-1 (36%)
AtCYP19-2	At2g21130	Cytosol (p^47, 48^)	—	BnCYP7-3 (59%), BnCYP27-1 (46%)
AtCYP19-3	At3g56070	Cytosol (p^47, 48^)	pathogen defence (inhibition of replication by binding to viral replication proteins (TBSV)), Ca^2+^ signalling, interaction with calmodulin (35–70 aa), sensitive to Cu^2+^ (implying redox regulation)^84, 91^	BnCYP19-1 (91%), BnCYP19-2 (90%), BnCYP5 (48%)
AtCYP19-4	At2g29960	Cytosol+SP (e^92, 93^)	cell polarity (regulates GNOM activity during embryogenesis), up-regulated after salt and cytokinin treatment^48, 92, 93^	BnCYP21-5 (93%), BnCYP21-3 (92%), BnCYP12-1 (56%), BnCYP14-4 (52%)
AtCYP20-1	At5g58710	SP (p^47, 48^)	UPR (unfolded protein response) in the ER, up-regulated upon ER stress, interaction with PP2A a component of multiple signalling pathways (e.g. auxin transport, growth response)^94, 95^	BnCYP21-9 (97%), BnCYP21-4 (92%), BnCYP21-6 (92%)
AtCYP20-2	At5g13120	TL (e^96, 97^)	down-regulated after pathogen treatment and up-regulated after light treatment, NAD(P)H dehydrogenase complex subunit, PPIase activity induced by oxidative stress, plant development (interacting with BZR1, a transcription factor responding to brassinosteroids (hormone signalling)), general protein folding catalyst in the TL^48, 62, 63, 97–100^	BnCYP27-3 (86%), BnCYP27-5 (86%), BnCYP7-2 (51%)
AtCYP20-3	At3g62030	Stroma (e^96, 97, 101^)	light and oxidative stress, redox regulation suggested (interacts with peroxiredoxins PrxA and PrxB), cysteine biosynthesis (interaction with SAT1 (serine acetyltransferase)), JA signalling, binds to JA and OPDA, interacts with *A. tumefaciens* VirD2 protein^38, 40, 85, 102–104^	BnCYP27-2 (89%), BnCYP27-4 (89%), BnCYP28-1 (85%), BnCYP28-2 (85%), BnCYP14-5 (81%), BnCYP24-3 (79%)
AtCYP21-1	At4g34960	SP (p^47, 48^)	—	BnCYP24-5 (95%), BnCYP24-6 (95%), BnCYP24-4 (85%), BnCYP24-7 (83%)
AtCYP21-2	At3g55920	SP (p^47, 48^)	water stress^105^	BnCYP24-1 (92%), BnCYP24-2 (92%)
AtCYP21-3	At2g47320	Mitochondria (p^47, 48^)	—	BnCYP25-4 (83%), BnCYP25-5 (83%)
AtCYP21-4	At3g66654	Mitochondria (p^47, 48^)	down-regulated after dark treatment^48^	BnCYP26-3 (89%), BnCYP26-1 (88%), BnCYP146 (87%), BnCYP26-5 (86%)
AtCYP22	At2g38730	Cytosol (p^47, 48^)	—	BnCYP21-2 (93%), BnCYP21-7 (93%), BnCYP21-8 (92%), BnCYP22-2 (92%)
AtCYP23	At1g26940	SP (p^47, 48^)	—	BnCYP25-3 (92%), BnCYP25-2 (87%), BnCYP25-1 (86%)
AtCYP26-1	At3g22920	Cytosol (p^47, 48^)	—	—
AtCYP26-2	At1g74070	TL (p^47, 48, 106^)	down-regulated after sucrose treatment^48^	BnCYP34-1 (84%), BnCYP34-2 (83%)
AtCYP28	At5g35100	TL (e^96, 106^)	down-regulated after dark and high CO_2_ treatment^48^	BnCYP30-1 (86%), BnCYP30-2 (85%)
AtCYP37	At3g15520	TL (e^96, 106^)	down-regulated after dark treatment^48^	BnCYP49 (87%), BnCYP50 (87%)
AtCYP38	At3g01480	TL (e^96, 97^)	no PPIase activity in the TL, PsbQ-like domain, photo system II: folding of subunits and assembly, down-regulated after dark treatment and up-regulated after light treatment^48, 61–65^	BnCYP47-2 (86%), BnCYP47-3 (84%), BnCYP22-1 (69%), BnCYP31 (51%)
AtCYP40	At2g15790	Cytosol (p^47, 48^)	interaction with HSP90, AGO1, miRNA156, regulating RISC complex, regulation of vegetative phase change^56, 57, 107^	BnCYP40-2 (93%), BnCYP40-1 (92%)
AtCYP57	At4g33060	Cytosol/Nucleus (p^47, 48^)	pathogen defence (callose accumulation), up-regulated after *P. syringae* infection, RNA-interacting region^30, 47, 48^	BnCYP55 (86%), BnCYP52 (80%)
AtCYP59	At1g53720	Nucleus (e^67^)	cyclophilin-RNA interacting protein (CRIP), Zinc finger motif, pre-mRNA processing, transcription (modulates RNA polymerase II activity)^32, 36, 67^	BnCYP12-2 (97%), BnCYP112 (85%), BnCYP10-2 (69%)
AtCYP63	At3g63400	Nucleus (p^47, 48^)	may be involved in RNA metabolism^47^	BnCYP62 (65%), BnCYP67 (65%)
AtCYP65	At5g67530	Cytosol (p^47, 48^)	suggested to be involved in the ubiquitin degradation pathway^47^	BnCYP65-1 (92%), BnCYP65-2 (92%), BnCYP26-2 (33%), BnCYP47-1 (27%), BnCYP37-1 (26%), BnCYP37-2 (26%)
AtCYP71	At3g44600	Nucleus (e^35^)	plant development, gene expression (histone modification, chromatin assembly), down-regulated after auxin treatment and after knox (transcription factors) overexpression^35, 48, 60^	BnCYP70-1 (91%), BnCYP70-2 (91%)
AtCYP95	At4g32420	Nucleus (p^47, 48^)	may be involved in RNA metabolism^47^	BnCYP86-1 (69%), BnCYP86-2 (67%)

*Localization was either p = predicted (SP, secretory pathway; TL, thylakoid lumen) or e = experimentally proven as described in the references.

**Figure 2:**
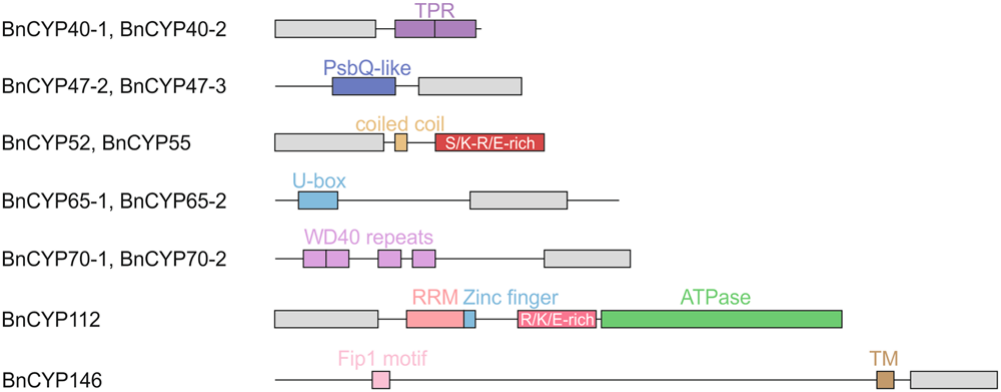
Domain structure of multi-domain *B. napus* cyclophilins. While most of the CYPs are single-domain proteins, 12 BnCYPs possess additional domains for specific tasks. CLDs are represented by a grey box. RRM = RNA recognition motif, TPR = tetratricopeptide repeat, TM = transmembrane domain.

For 12 *B. napus* CYPs additional domains are predicted which promote diverse capabilities like interaction (protein-protein, protein-DNA, protein-RNA) and modification (ubiquitination) (Fig. 2). These seem to be conserved among *Brassicaceae*, since similar domain structures exist in the corresponding *A. thaliana* homologs.

The MD CYPs BnCYP40-1 and BnCYP40-2 are characterized by a tetratricopeptide repeat (TPR) motif with two TPRs, each 34 amino acids long. Such motifs mediate protein-protein interactions and can thereby assist in the assembly of multi-protein complexes. BnCYP40-1 and BnCYP40-2 show a high degree of sequence identity (92 and 93%, respectively) to AtCYP40 (Table 2). A cytoplasmic interaction partner of AtCYP40 is HSP90, which mediates its recruitment to an intermediate RISC complex by its TPR motifs^56, 57^. Thereby, AtCYP40 forms, together with HSP90, a complex with AGO1 and a small RNA duplex before the mature RISC complex consisting of AGO1 and a siRNA or miRNA strand is formed^56^. The mechanism of CYP40-HSP90 binding is conserved between different species. Besides plants, human HSP90 is also known to bind to hCYP40, the human AtCYP40 homolog^58^. Thus, a similar function for the highly identical CYPs BnCYP40-1 and BnCYP40-2 can be assumed.

Other CYPs showing classical protein-protein interaction domains are BnCYP70-1 and BnCYP70-2, both containing four tryptophan-aspartic acid (WD40) repeats. These are short structural motifs typically forming a four stranded anti-parallel β-sheet. Multiple copies build a circular β-propeller structure promoting protein-protein interactions. Both show 91% sequence identity to their homolog in Arabidopsis, AtCYP71 (Table 2), which is located in the nucleus and functions in the regulation of gene expression and organogenesis. The WD40 domain enables AtCYP71 to associate with histone H3 affecting its methylation^35^. Data from yeast PPIases provide evidence that they are responsible for histone modification by utilizing the peptidyl-prolyl bond isomerisation as a molecular switch for transcription regulation^59^. Furthermore, AtCYP71 was shown to interact with FAS1 (a subunit of Chromatin Assembly Factor-1) and LHP1 (a heterochromatin protein)^60^, suggesting that AtCYP71 is involved in histone modification and chromatin assembly.

BnCYP47-2 and BnCYP47-3 both contain a putative PsbQ-like domain and have only small differences in their N-terminal amino acid sequences. Their closest homolog is AtCYP38 with 86 and 84% sequence identity (Table 2), which also contains a PsbQ-like domain. This domain is typical for proteins localized in the chloroplast, but its function is mostly unknown. AtCYP38 is the only Arabidopsis CYP for which a crystal structure is available. This revealed the additional PsbQ-like domain^61^. AtCYP38 is experimentally assigned to the chloroplast thylakoid lumen, does not show any PPIase activity^62, 63^, and plays a critical role in the assembly and maintenance of photosystem II^61, 64, 65^. It is suggested to interact with the E-loop of chlorophyll protein47 (CP47), a component of the photosystem II (PSII) complex^61^, via its CLD. Furthermore it is proposed to be responsible for proper folding and insertion of D1 and CP43, both components of PSII^65^.

There are also two BnCYPs showing classical domains for protein modification. BnCYP65-1 and BnCYP65-2 both contain a Zinc finger U-box motif. Therefore, they might be involved in the ubiquitin degradation pathway.

Four BnCYPs contain sequences characteristic for RNA-interacting proteins. BnCYP52 and BnCYP55 both possess a coiled coil domain. This domain represents a structural motif with coiled α-helices. Proteins containing such a domain can, for example, be transcription factors involved in the regulation of gene expression. The respective *A. thaliana* homolog is AtCYP57, which shows, besides a predicted coiled coil domain, also an S/K-R/E-rich region^47^. Sequence comparisons of BnCYP52, BnCYP55 and AtCYP57 revealed a high sequence identity in the AtCYP57 S/K-R/E-rich region (data not shown). This region is supposed to mediate the interaction with ribonucleoproteins^66^.

BnCYP112 contains an RNA recognition motif (RRM), a Zinc finger motif (CCHC-type), a positively charged region (arginine, lysine, glutamate) and an actin-like ATPase domain. Besides AtCYP59 in *A. thaliana*, homologs to BnCYP112 can be found in different species like *P. tetraurelia*, *S. pombe*, *C. elegans*, *D. melanogaster*, and *H. sapiens* ^32^. This group of proteins is called CRIPs, for cyclophilin-RNA interacting proteins^32^. Characteristic for this group of proteins is an RRM in addition to the CLD. Nevertheless, CRIPs differ in their C-terminal region. AtCYP59 and BnCYP112 are the only members containing a Zinc finger motif. AtCYP59 interacts with the C-terminal domain of the largest subunit of RNA polymerase II and thereby influences its phosphorylation state. Furthermore, binding of an RNA transcript decreases PPIase activity of AtCYP59. This might modulate RNA polymerase II activity^36^. Therefore, it is suggested that AtCYP59 connects pre-mRNA processing and transcription^36, 67^. Moreover, BnCYP112 is the only homolog of all CRIPs showing an additional actin-like ATPase domain.

BnCYP146 is the largest CYP found in oilseed rape. It contains a transmembrane domain and a Fip1 motif. In yeast, the Fip1 protein ensures the polyadenylation of mRNAs by interacting with poly(A) polymerase. There is no *A. thaliana* homolog showing the same domain structure as BnCYP146, but its CLD is closely related to AtCYP21-4 (Table 2).

### Subcellular localization and phylogenetic relationships

**Figure 3:**
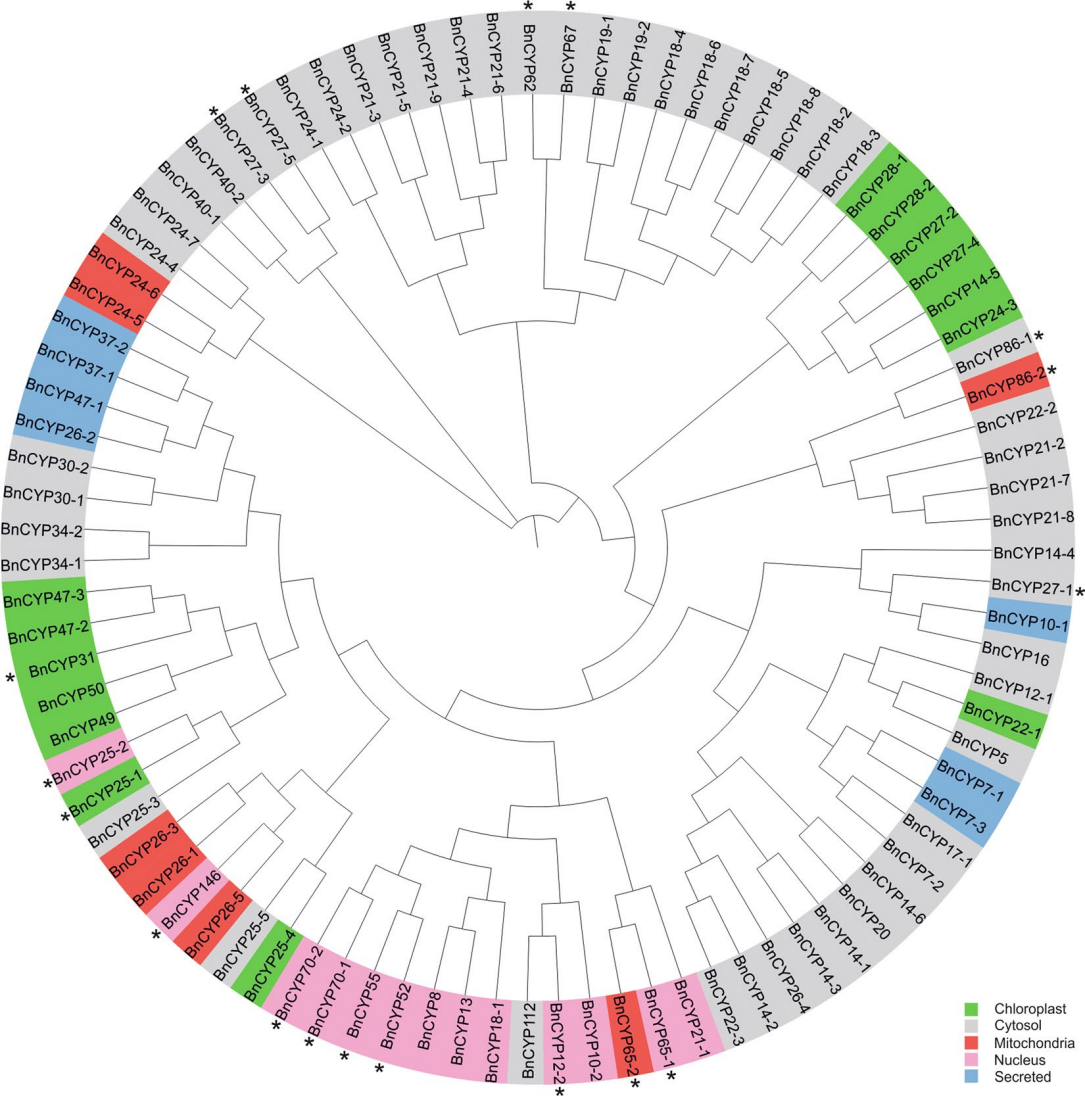
Phylogenetic tree of *B. napus* cyclophilins. The phylogenetic analysis was based on a sequence alignment by ClustalOmega and constructed as described in Materials and Methods with ignored branch lengths. As indicated by colour code, this protein family is distributed over all intercellular organelles. In addition, some BnCYPs possess putative nuclear localization signals (marked by *).

The phylogenetic analysis of the full length amino acid sequences revealed clusters of proteins with high sequence similarity (Fig. 3). Often, these groups possess the same additional domains, consistent subcellular localization, and the same *A. thaliana* CYP homolog.

As previously described for other organisms (e.g. *A. thaliana* homologs, see Table 2), *B. napus* CYPs are targeted to all intercellular organelles. At least 50 BnCYPs are predicted to be located in the cytosol. This result is comparable to the subcellular distribution of the *A. thaliana* CYPs where 14 of 29 are either predicted or experimentally proven to be located in the cytosol (Table 2). Furthermore, 14 BnCYPs are predicted to be located in chloroplasts, 7 in mitochondria, 13 in the nucleus, and 7 are secreted (summarized in Fig. 3, for a detailed prediction see Supplementary Table S1). In addition, some BnCYPs contain nuclear localization signals (NLS). Proteins predicted to be cytosolic or nuclear which possess NLS might relocate between both compartments.

### Genomic distribution of cyclophilins

**Figure 4:**
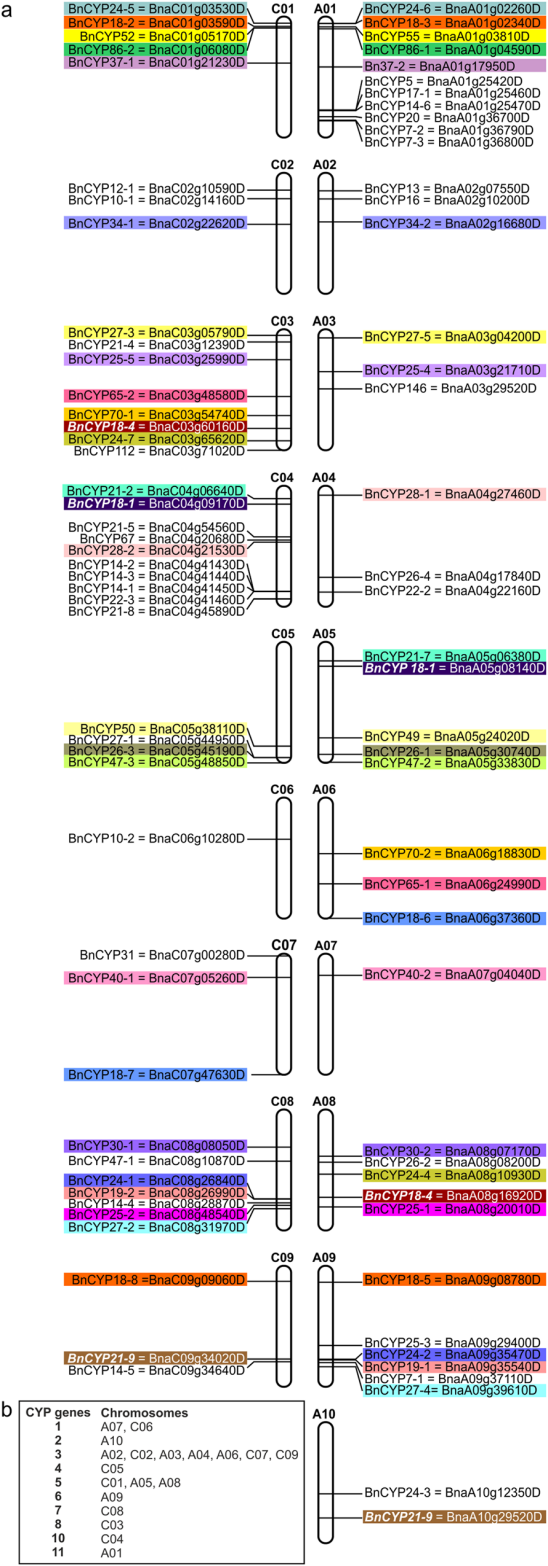
Genomic distribution of *cyclophilin* genes on *B. napus* chromosomes. (**a**) Chromosomal locations of *BnCYP* genes are indicated based on the information provided by the *Brassica napus* Genome Browser. Highlighted by identical colours are *BnCYP* genes which encode protein isoforms. Genes encoding identical proteins are written in italics and white colour. (**b**) Summary of the number of *BnCYP* genes encoded on each chromosome.

*CYP* genes are distributed on all 19 chromosomes of the *B. napus* genome (Fig. 4a). There is no chromosome that is not encoding any *CYP*. Chromosomes A07 and C06 contain only one *CYP* each. Chromosome A01 has the largest number with 11 *CYP* genes. All other chromosomes contain between 2 and 10 *CYP* genes (Fig. 4b). The 4 *CYP* genes *BnaAnng41240D* (*BnCYP12-2*), *BnaAnng15590D* (*BnCYP21-3*), *BnaAnng17350D* (*BnCYP21-6*), and *BnaAnng12550D* (*BnCYP62*) were mapped to the A chromosomes, but without a detailed information regarding the exact chromosomal location. Likewise, the 4 *CYP* genes *BnaCnng42430D* (*BnCYP8*), *BnaCnng08980D* (*BnCYP21-1*), *BnaCnng32070D* (*BnCYP22-1*), and *BnaCnng32180D* (*BnCYP26-5*) were mapped to the C chromosomes, but again without a detailed information regarding the exact chromosomal location. The A chromosomes contain 42 *CYP* genes and 4 not fully assigned genes, and the C chromosomes contain 44 *CYP* genes and 4 not fully assigned genes. Thus, both chromosome sets (A and C), which originate from the *B. rapa* and *B. oleracea* genome, contain a similar number of *CYP* genes.

Interestingly, there are three CYP proteins that are each encoded by two of the six following genes *BnaA05g08140D* and *BnaC04g09170D* (BnCYP18-1), *BnaA08g16920D* and *BnaC03g60160D (*BnCYP18-4), or *BnaA10g29520D* and *BnaC09g34020D* (BnCYP21-9). Thus, one copy of these genes occurs on each of the chromosomal sets. Alignments of these *CYP* genes from the A and respective C chromosome showed slightly different nucleotide sequences. Nevertheless, these encode identical amino acid sequences (data not shown).

Due to the two progenitor chromosomal sets, several *CYP* genes encode proteins with high sequence homologies. Most often these occur as pairs, each gene originating from one progenitor chromosomal set. They are highlighted with identical colours on the A and C chromosomes in Fig. 4 and are from now on called isoforms. Some of them are located on the respective A and C chromosome, others are spread on different chromosomes, probably due to chromosomal rearrangements of the *B. napus* genome (as recently described by Cheng *et al*.^68^).

In summary, the allopolyploidy of oilseed rape results in two isoforms of many proteins. These show high sequence identities on the nucleotide and amino acid levels and originate from either the A or C genome. Due to their similarity, they cluster in the phylogenetic tree and often the same localization is predicted (Fig. 3). They might either possess the ability to replace each other or might be specialized for certain tasks and interaction partners.

One of the biggest groups of isoforms among the BnCYPs is the group of 18 kDa proteins. These contain a full-length CLD with a high degree of conservation, a cytosolic localization, and can be further subdivided by their homology. The phylogenetic tree reveals isoform pairs: BnCYP18-2 and BnCYP18-3, BnCYP18-5 and BnCYP18-8, and BnCYP18-6 and BnCYP18-7 (Fig. 3). Each pair has a representative gene on chromosome A and C, respectively (Fig. 4a). Interestingly, BnCYP18-4 which is closely related to the pair BnCYP18-6/18-7 is encoded by two genes located on C03 and A08 that might derive from a gene duplication event. The *A. thaliana* homologs of the described 18 kDa isoforms are AtCYP18-3, AtCYP18-4, and AtCYP19-1 which are shown to be important for various processes from plant growth to pathogen defence (summarized in Table 2). In contrast to the other 18 kDa BnCYPs, BnCYP18-1 is the only 18 kDa CYP with introns and without the prominent insertion between α-helix-I and β-sheet-III. Moreover, its localization is predicted to be nuclear instead of cytosolic (Fig. 3).

### Expression analysis of the predicted BnCYPs on transcript and protein levels

**Figure 5:**
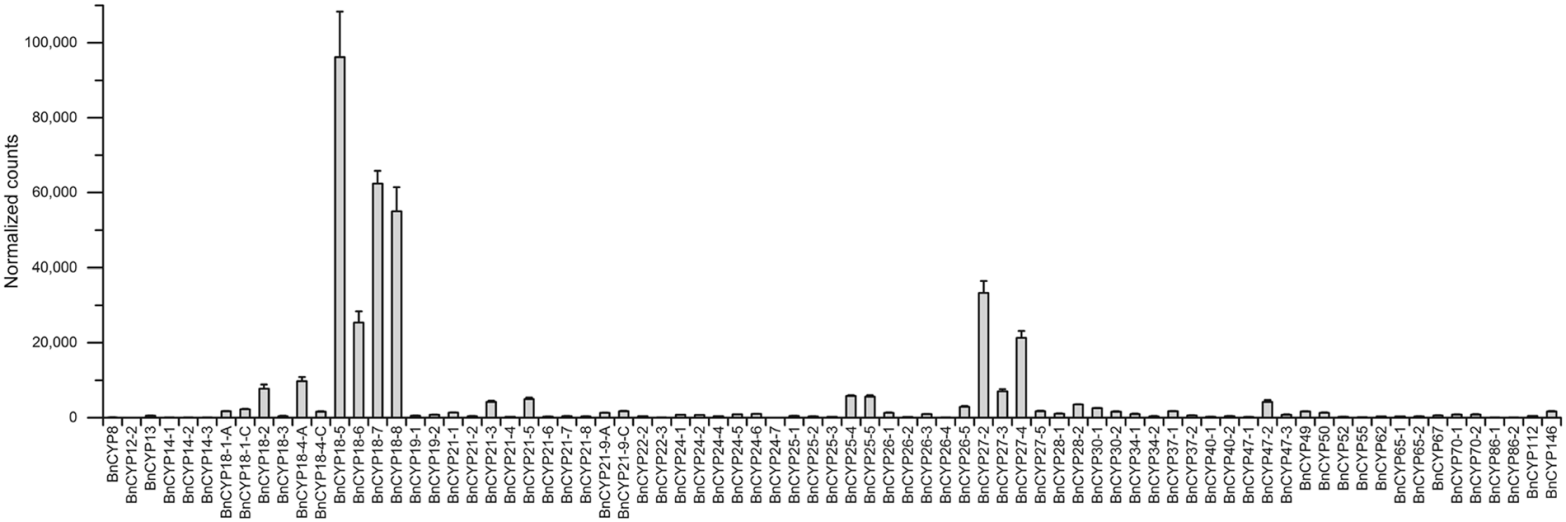
Expression pattern of *BnCYP* genes from leaf material. In case of two genes encoding the same BnCYP protein, the transcript is abbreviated by its BnCYP name with -A or -C depending on the chromosomal location of the gene, respectively. Raw data are provided in Supplementary Table S2.

To investigate the mRNA expression of the bioinformatically predicted *CYP* genes, RNA-Seq of RNA isolated from leaves was performed. This approach revealed 77 expressed *BnCYPs* under the applied conditions (summarized in Table 3). The expression pattern indicates a large variance in the abundance of *BnCYP* transcripts (Fig. 5, for raw data see Supplementary Table S2). The results thus show that most of the predicted *CYP* genes are indeed transcribed. Transcripts for *CYP* genes not detected in this study could be expressed in other tissues, specific cell types, or under different experimental conditions.

**Table 3:** Identified *B. napus* cyclophilins by RNA-Seq and LC-MS/MS.

Name	Gene Name	BnCYPs identified by RNA-Seq	BnCYPs identified by LC-MS/MS	
			Leaf	Phloem
BnCYP5	BnaA01g25420D			
BnCYP7-1	BnaA09g37110D			
BnCYP7-2	BnaA01g36790D			
BnCYP7-3	BnaA01g36800D			
BnCYP8	BnaCnng42430D	x		
BnCYP10-1	BnaC02g14160D			
BnCYP10-2	BnaC06g10280D			
BnCYP12-1	BnaC02g10590D			
BnCYP12-2	BnaAnng41240D	x		
BnCYP13	BnaA02g07550D	x		x
BnCYP14-1	BnaC04g41450D	x		
BnCYP14-2	BnaC04g41430D	x		
BnCYP14-3	BnaC04g41440D	x		
BnCYP14-4	BnaC08g28870D			
BnCYP14-5	BnaC09g34640D			
BnCYP14-6	BnaA01g25470D			
BnCYP16	BnaA02g10200D			
BnCYP17-1	BnaA01g25460D			
BnCYP18-1	BnaA05g08140D	x		x
	BnaC04g09170D	x		
BnCYP18-2	BnaC01g03590D	x		x
BnCYP18-3	BnaA01g02340D	x		
BnCYP18-4	BnaC03g60160D	x	x	x
	BnaA08g16920D	x		
BnCYP18-5	BnaA09g08780D	x	x	x
BnCYP18-6	BnaA06g37360D	x		x
BnCYP18-7	BnaC07g47630D	x		x
BnCYP18-8	BnaC09g09060D	x		
BnCYP19-1	BnaA09g35540D	x		x
BnCYP19-2	BnaC08g26990D	x	x	x
BnCYP20	BnaA01g36700D			
BnCYP21-1	BnaCnng08980D	x		x
BnCYP21-2	BnaC04g06640D	x		
BnCYP21-3	BnaAnng15590D	x	x	x
BnCYP21-4	BnaC03g12390D	x		x
BnCYP21-5	BnaC04g54560D	x		
BnCYP21-6	BnaAnng17350D	x		
BnCYP21-7	BnaA05g06380D	x		
BnCYP21-8	BnaC04g45890D	x		
BnCYP21-9	BnaA10g29520D	x		x
	BnaC09g34020D	x		
BnCYP22-1	BnaCnng32070D			
BnCYP22-2	BnaA04g22160D	x		x
BnCYP22-3	BnaC04g41460D	x		
BnCYP24-1	BnaC08g26840D	x	x	
BnCYP24-2	BnaA09g35470D	x		
BnCYP24-3	BnaA10g12350D			
BnCYP24-4	BnaA08g10930D	x		
BnCYP24-5	BnaC01g03530D	x		
BnCYP24-6	BnaA01g02260D	x		
BnCYP24-7	BnaC03g65620D	x		
BnCYP25-1	BnaA08g20010D	x		
BnCYP25-2	BnaC08g48540D	x		
BnCYP25-3	BnaA09g29400D	x		
BnCYP25-4	BnaA03g21710D	x		
BnCYP25-5	BnaC03g25990D	x		
BnCYP26-1	BnaA05g30740D	x		
BnCYP26-2	BnaA08g08200D	x		
BnCYP26-3	BnaC05g45190D	x		
BnCYP26-4	BnaA04g17840D	x		
BnCYP26-5	BnaCnng32180D	x		
BnCYP27-1	BnaC05g44950D			
BnCYP27-2	BnaC08g31970D	x	x	x
BnCYP27-3	BnaC03g05790D	x	x	x
BnCYP27-4	BnaA09g39610D	x	x	x
BnCYP27-5	BnaA03g04200D	x		
BnCYP28-1	BnaA04g27460D	x	x	x
BnCYP28-2	BnaC04g21530D	x		x
BnCYP30-1	BnaC08g08050D	x	x	
BnCYP30-2	BnaA08g07170D	x		
BnCYP31	BnaC07g00280D			
BnCYP34-1	BnaC02g22620D	x	x	
BnCYP34-2	BnaA02g16680D	x		
BnCYP37-1	BnaC01g21230D	x		
BnCYP37-2	BnaA01g17950D	x		
BnCYP40-1	BnaC07g05260D	x		
BnCYP40-2	BnaA07g04040D	x		
BnCYP47-1	BnaC08g10870D	x		
BnCYP47-2	BnaA05g33830D	x	x	
BnCYP47-3	BnaC05g48850D	x		
BnCYP49	BnaA05g24020D	x	x	
BnCYP50	BnaC05g38110D	x		
BnCYP52	BnaC01g05170D	x		
BnCYP55	BnaA01g03810D	x		
BnCYP62	BnaAnng12550D	x	x	
BnCYP65-1	BnaA06g24990D	x		
BnCYP65-2	BnaC03g48580D	x		
BnCYP67	BnaC04g20680D	x		
BnCYP70-1	BnaC03g54740D	x		
BnCYP70-2	BnaA06g18830D	x		
BnCYP86-1	BnaA01g04590D	x		
BnCYP86-2	BnaC01g06080D	x		x
BnCYP112	BnaC03g71020D	x		
BnCYP146	BnaA03g29520D	x

In the analysed leaf sample, the 18 kDa and 27 kDa family members belong to the strongest expressed ones, with *BnCYP18-5* showing the highest read count. Interestingly, some isoform pairs had differing read counts, e.g. *BnCYP18-2*/*BnCYP18-3*, *BnCYP18-4*/*BnCYP18-6*/*BnCYP18-7, BnCYP27-3*/*BnCYP27-5*.

Since CYPs are a prominent protein class in the phloem long-distance transport system of higher plants including oilseed rape with a potential function as a molecular chaperone important for protein long-distance transport^2, 41^, we performed LC-MS/MS analysis of phloem protein extract and compared the CYP protein profile to that of leaf extract. Table 3 summarizes the BnCYPs identified from leaf and phloem protein extracts. In total, 26 BnCYPs could be detected at the protein level under the applied conditions with six being unique to leaves and 12 to phloem exudate. Since sieve elements do not have a functional transcription machinery, RNA-Seq was not performed with phloem samples. However, phloem samples contain a specific set of mobile RNAs that have in part been implicated with long-distance signalling^43^, but this was not the subject of the present study.

**Figure 6:**
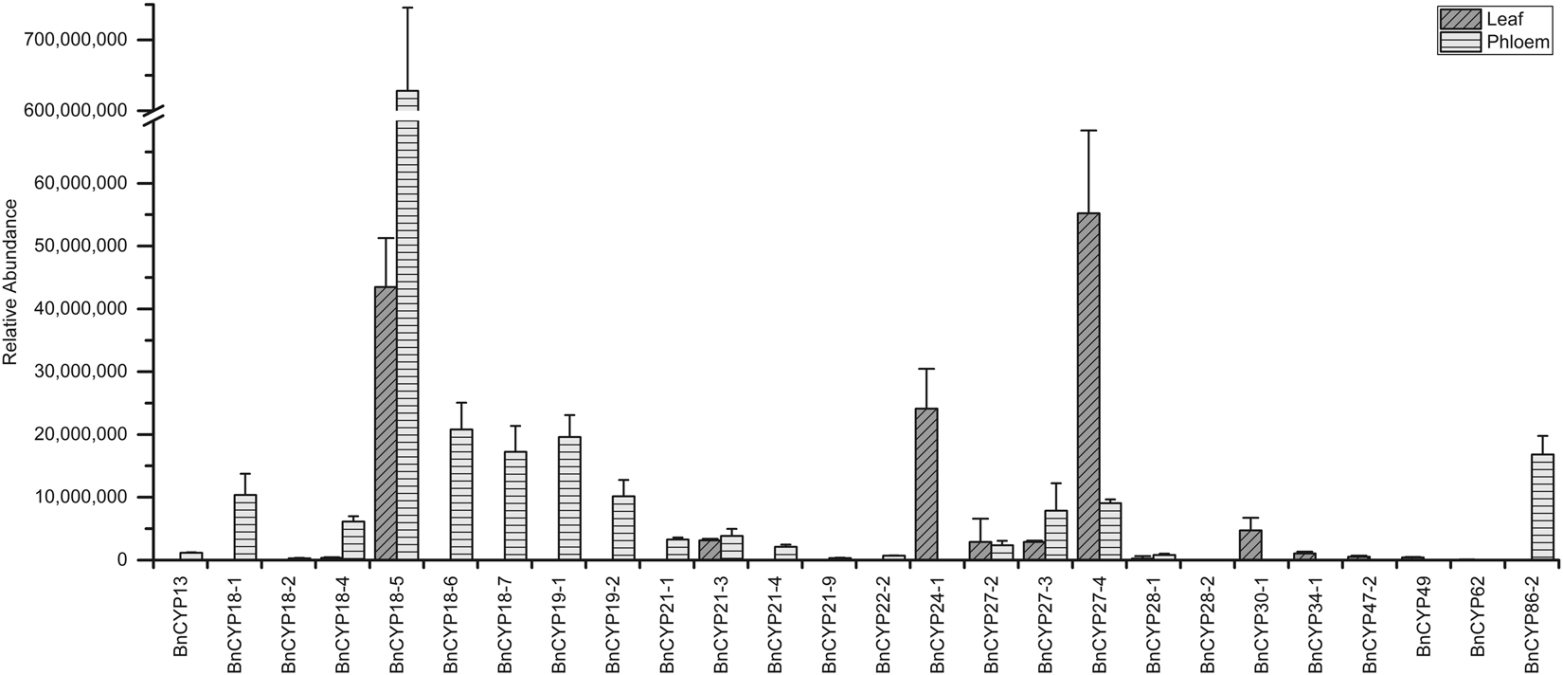
Relative abundance of leaf and phloem BnCYP proteins. The relative abundance of each BnCYP refers to the abundance of its unique peptides either from leaf extract (n = 3) or phloem sap (n = 4) samples.

Most of the identified BnCYP proteins are predicted to be localized in the cytosol, but some potentially chloroplastic, mitochondrial and nuclear BnCYPs were found as well. Besides several SD CYPs, also one MD CYP, the putative chloroplastic protein BnCYP47-2, could be identified in leaf extract. The analysis of phloem sap revealed a low molecular weight CYP, BnCYP13, which is suggested to contain only a partial CLD. Figure 6 shows the abundance of the identified CYP proteins in the two examined organs (more details in Supplementary Tables S3, S4, S5, S6). Whereas BnCYP18-5, BnCYP24-1 and BnCYP27-4 were the most abundant CYP proteins in leaf extract, only BnCYP18-5 was the most abundant one in phloem sap with a considerably higher relative abundance observed than for the other identified BnCYP proteins from this compartment. Interestingly, the most abundant CYP protein in both compartments is BnCYP18-5. The finding of so many CYPs in the phloem transport system suggests an essential function of this protein family in this compartment, probably in protein transport or long-distance signalling.

## Conclusions

Cyclophilins are ubiquitous proteins that constitute a multigene family in higher organisms. Exceptionally high numbers of CYPs have been found in plants, underlining their essential importance in many essential physiological processes. However, the physiological roles of most CYPs in plants are not well understood.

The present study applied bioinformatic tools for a genome-wide identification of CYPs in the important oil crop *B. napus*. Sequence similarity searches with known Arabidopsis CYPs, sequence alignments and CLD prediction identified a surprisingly high number of 94 CYP-coding genes. Therefore, *B. napus* contains the highest number of CYPs known so far. As in other plants, CYPs are predicted to be localized in all compartments, most of them being probably cytosolic. Most BnCYPs are single-domain proteins.

Transcriptome analysis confirmed the expression of 77 distinct CYPs in the *B. napus* cultivar ‘Drakkar’ from leaf material under normal growth conditions. The occurrence of 26 BnCYP proteins was confirmed by LC-MS/MS analysis. It is likely that additional CYPs are expressed on transcript and protein levels in different cell types, plant parts, or under different environmental conditions. Interestingly, 12 BnCYPs were exclusively found in phloem samples and not in leaf extract supporting a fundamental and specific role in this specialized compartment.

Future studies must now focus on the functional characterization of the high number of CYPs in *B. napus* in order to better understand the diverse roles of CYPs in oilseed rape and in plant biology in more general. In this regard, elucidating the role(s) of the phloem CYPs in protein refolding and long-distance signalling will be of special interest.

## Materials and Methods

### Sequence analysis

*Brassica napus* CYPs were identified by BLASTp of the *Arabidopsis thaliana* CYP18-1 (At1g01940) and CYP19-1 (At2g16600) against the *B. napus* genome sequence database^51^ deposited at NCBI (http://www.ncbi.nlm.nih.gov/)^69^. BLASTp searches with the remaining AtCYPs revealed the same BnCYPs as already identified. Amino acid and cDNA sequences were obtained by the European Nucleotide Archive (http://www.ebi.ac.uk/ena/data/view/PRJEB5043). To identify *A. thaliana* homologs for the individual BnCYPs, the amino acid sequences of BnCYPs were used as queries for a BLAST search on UniProtKB (http://www.uniprot.org/)^70^.

All identified proteins were analyzed for the presence of a CLD and potential additional domains with InterPro (http://www.ebi.ac.uk/interpro/)^71^ and drawn by CorelDRAW. The theoretical isoelectric point was determined by the ProteinProspector Tool MS-Digest (http://prospector.ucsf.edu/prospector/cgi-bin/msform.cgi?form=msdigest), subcellular localization was predicted by LocTree3 (https://rostlab.org/services/loctree3/)^72^ and nuclear localization signals (NLS) by using the NLS mapper (http://nls-mapper.iab.keio.ac.jp/cgi-bin/NLS_Mapper_form.cgi)^73^.

Chromosome mapping of the CYPs was performed using the *Brassica napus* Genome Browser (http://www.genoscope.cns.fr/brassicanapus/cgi-bin/gbrowse/colza/) available by Genoscope - Centre National de Séquençage and redrawn by CorelDRAW.

### Protein sequence alignment and phylogenetic analysis

Sequences were aligned using ClustalOmega (http://www.ebi.ac.uk/Tools/msa/clustalo/)^74^ and displayed with Jalview 2.9.0b2^75^. The secondary structure annotation is based on the structure of the crystallized human CYPA (4N1M.pdb).

The phylogenetic tree was calculated with ClustalW2 Phylogeny (http://www.ebi.ac.uk/Tools/phylogeny/clustalw2_phylogeny/) by using the multiple alignment from ClustalOmega, and subsequently processed with iTOL (http://itol.embl.de/)^76, 77^.

### Plant material and growth conditions

*Brassica napus* cultivar ‘Drakkar’ plants were grown in 19 cm pots on soil (LAT-Terra Standard P, Industrie-Erdenwerk Archut, Germany) in a glasshouse under controlled conditions with 70% humidity and a 16 h/8 h light/dark (day/night) and 22 °C/18 °C (day/night) cycle. Plants were watered once per day and fertilized with 2 g/l Osmocote Exact Standard High K (Scotts, the Netherlands).

### Expression profile of cyclophilins

Transcriptome data were generated by GAMAVIR, a tri-national research activity aiming at characterizing plant:virus interactions in rapeseed (ANR-13-KBBE-0005). PolyA RNA isolated from leaf disks of 6 weeks-old *Brassica napus* cultivar ‘Drakkar’ plants (n = 3) was sequenced using Illumina technology. Clean RNAseq reads were aligned against the re-sequenced *Brassica napus* cultivar ‘Drakkar’ genome using Tophat2 v2.0.13^78^ and counted with Samtools v1.1^79^.

### Protein extraction for proteomics

Phloem sap was collected as described previously by Giavalisco *et al*.^2^ at the inflorescence of oilseed rape plants before flowering. Phloem sap samples were collected on ice four times (n = 4), frozen in liquid nitrogen and stored at −80 °C until further processing. Proteins were precipitated in 4 volumes of 90% (v/v) acetone, 10% (v/v) methanol, 10 mM DTT over night at −20 °C. The precipitates were pelleted at 14,000 × g at 4 °C for 15 min, washed twice with 100% acetone and air-dried.

Leaf material was harvested from three different plants (n = 3) and for each sample 100 mg material was grinded in liquid nitrogen. Proteins were extracted with 800 µl extraction buffer (50 mM MOPS/pH 7.5, 5% glycerol, 0.55% PVPP, 0.5% Nonidet P-40, 5 mM L-ascorbic acid, 5 mM DTT, 1x protease inhibitor (cOmplete Protease Inhibitor Cocktail, Roche), 1x phosphatase inhibitor (PhosStop, Phosphatase Inhibitor Cocktail, Roche). Centrifuging at 14,000 × g at 4 °C for 15 min allowed the separation of soluble proteins from insoluble material. Proteins were precipitated as described above by acetone/methanol/DTT.

### Analysis of phloem sap and leaf proteins by LC-MS/MS

For the analysis of phloem proteins the extracted and precipitated phloem sap proteins were resuspended in extraction buffer (6 M urea, 2 M thiourea, 15 mM DTT, 2% CHAPS). Here, each protein pellet was dissolved in exactly the same volume deployed for the initial phloem sap precipitation. Once the proteins were in solution, samples were sonicated for 10 min in a sonication bath, followed by 30 min incubation on an orbital shaker (100 rpm) at room temperature. Solubilised proteins were centrifuged at 10,000 × g for 5 min and the protein concentration was determined from the collected supernatant.

For the analysis of leaf tissue proteins, the precipitated proteins were resuspended in sufficient protein extraction buffer to provide a final protein concentration of 2 µg/µl.

25 µg of phloem sap and 50 µg of leaf tissue protein extract were then digested in solution using a Trypsin/Lys-C mixture (Mass Spec Grade, Promega, Madison, WI, USA) according to the instruction manual. After the digestion, the samples were desalted using C18-stage tips as described in Rappsilber *et al*.^80^.

After the elution of the digested and desalted peptides from C18-stage tips, the samples were concentrated to near dryness in a SpeedVac and the peptide mixtures were reconstituted in 30 µl resuspension buffer (5% acetonitrile, 0.1% formic acid in water). 5 µl of this peptide mix was injected onto an Acclaim PepMap RSLC HPLC column (75 µm × 15 cm, Thermo Scientific) connected to the EASY-nLC 1000 system (Thermo Scientific). The eluting peptides were then analyzed on a Q Exactive Plus (Thermo Scientific, Bremen, Germany) high-resolution mass spectrometer.

The peptides were separated using a binary buffer system of 0.1% formic acid in water (Buffer A) and 60% acetonitrile containing 0.1% formic (Buffer B). The flow rate was adjusted to 300 nl/min. Peptides were eluted with on a linear gradient of 0–40% buffer B for 50 min followed by a linear gradient between 40–80% buffer B for additional 30 min. Peptides were analyzed in the mass spectrometer using one full scan (300–1600 m/z, R = 70,000 at 200 m/z), followed by up to fifteen data-dependent MS/MS scans (Top 15 approach) with higher-energy collisional dissociation (HCD) at a resolution of 17,500 at 200 m/z. Dynamic exclusion was set to 15 s.

Raw data were processed using the Progenesis QI for proteomics (Progenesis QI for Proteomics Version 3.0, Nonlinear Dynamics, Newcastle, UK) software and the protein sequences of all identified CYPs from *Brassica napus*. Protein identifications were filtered with a false discovery rate better than 1%, at least two peptides, one unique peptide and a score of 50.

## Electronic supplementary material

Supplementary Information
