# Supplementary material for: Bioinformatic and expression analysis of the Brassica napus L. cyclophilins
Source: Sci Rep. 2017 May 4;7:1514. doi: 10.1038/s41598-017-01596-5 (PMC5431436; doi:10.1038/s41598-017-01596-5)
Supplement: Supplementary file 1 — Supplementary Information [file 41598_2017_1596_MOESM1_ESM.doc]

# Supplementary data

# Article title: Bioinformatic and expression analysis of the *Brassica napus* L. cyclophilins

# All author names: Patrizia Hanhart^1^, Melanie Thieß^1^, Khalid Amari^2^, Krzysztof Bajdzienko^3^, Patrick Giavalisco^3^, Manfred Heinlein^2^ and Julia Kehr^1*^

1 = Molecular Plant Genetics, University Hamburg, Biocenter Klein Flottbek, Ohnhorststr. 18, 22609 Hamburg, Germany

2 = Université de Strasbourg, CNRS, IBMP UPR 2357, 12 rue du Général Zimmer, F-67000 Strasbourg, France

3 = Max-Planck-Institut für Molekulare Pflanzenphysiologie, Wissenschaftspark Potsdam-Golm, Am Mühlenberg 1, 14476 Potsdam, Germany

* = corresponding author;

**Corresponding author(s) details**: Prof. Dr. Julia Kehr, Molecular Plant Genetics, University Hamburg, Biocenter Klein Flottbek, Ohnhorststr. 18, 22609 Hamburg, Germany;

**Corresponding author email:** julia.kehr@uni-hamburg.de


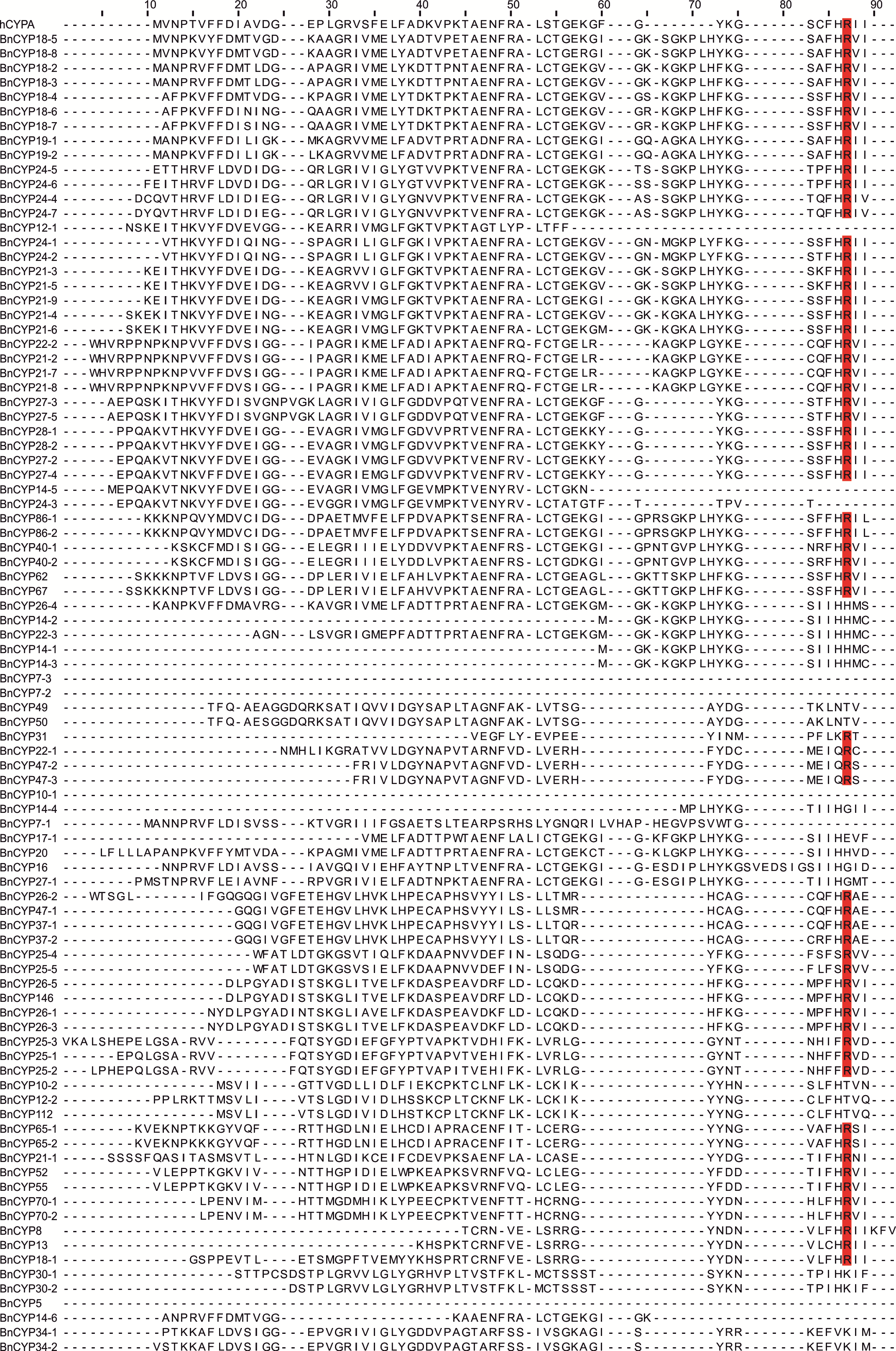


**
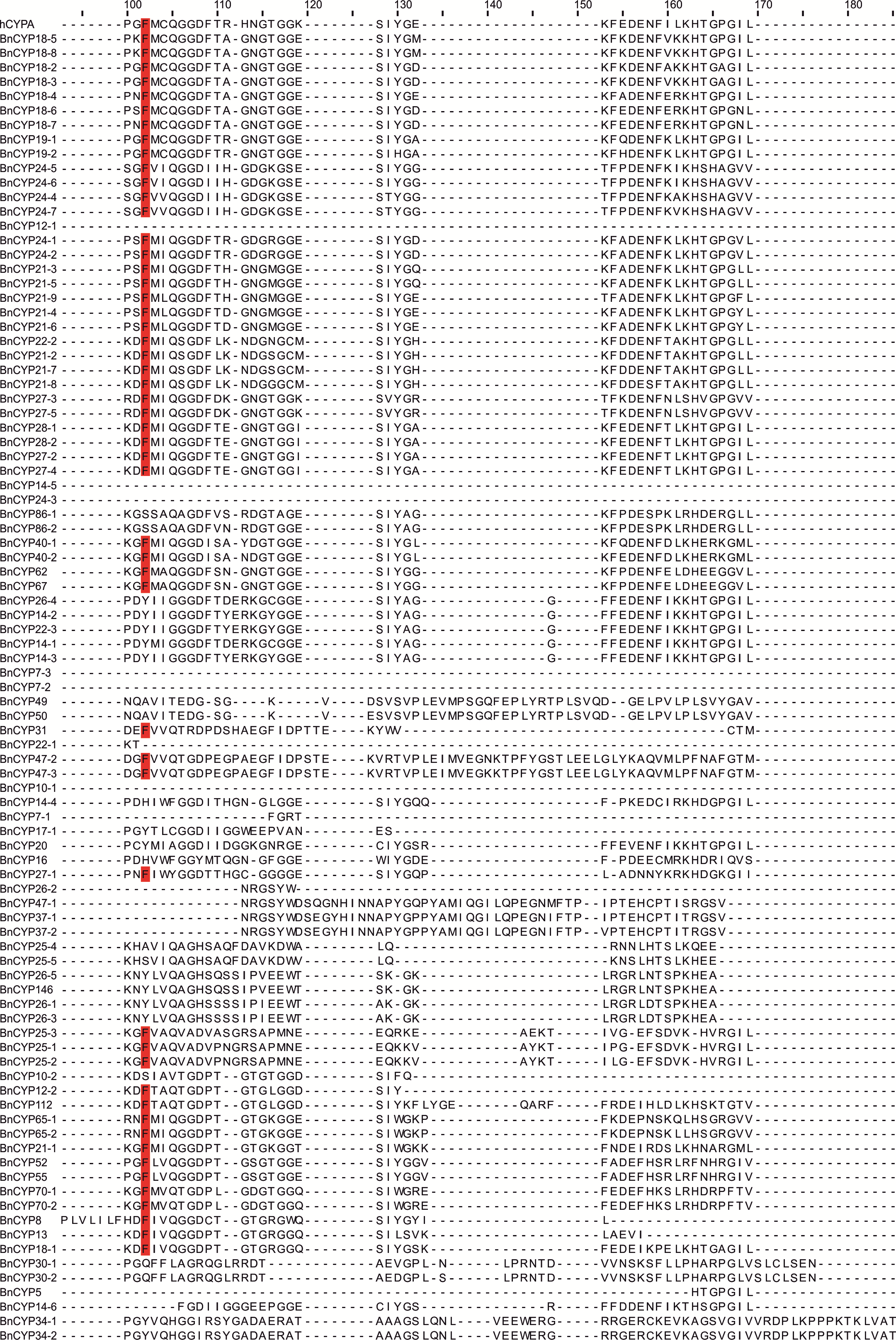
**

**
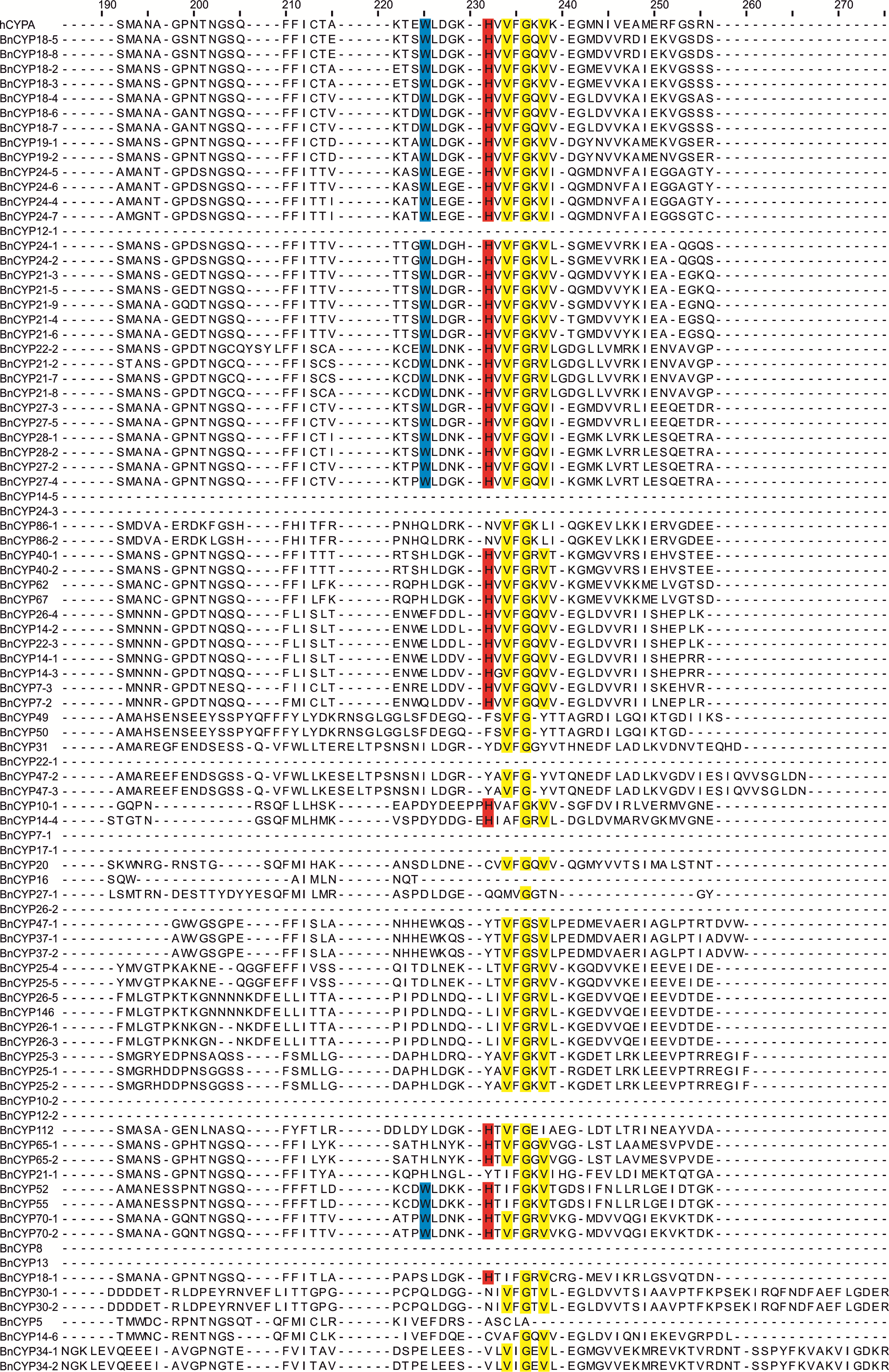
**

#
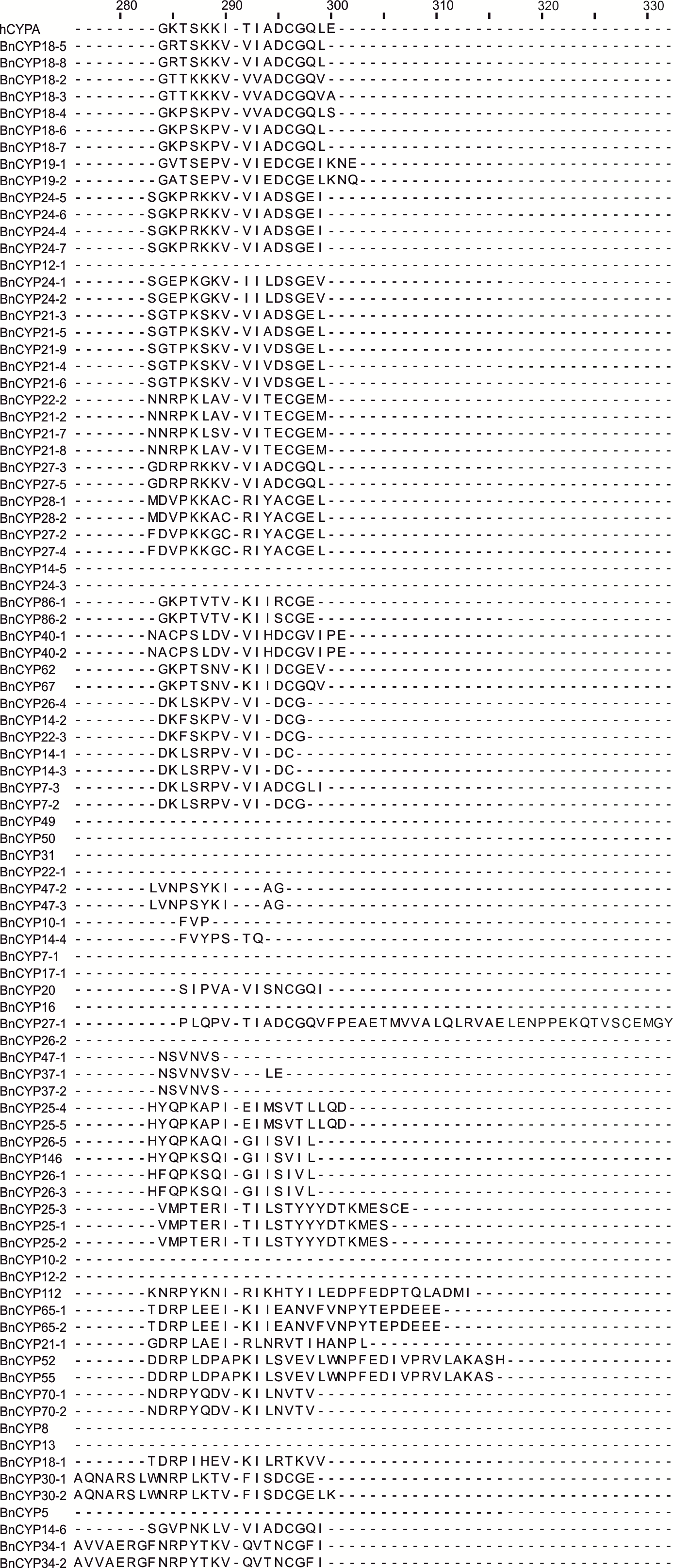
Supplementary Figure S1. Multiple alignment of the CLD protein sequences of the *B. napus* CYP family (whole protein sequences were cropped to the predicted CLD sequence according to Table 1). Conserved residues are highlighted: yellow = amino acids of the VXGXV motif discovered in Arabidopsis CYPs; red = amino acids important for PPIase activity; blue = amino acids required for CsA binding.

**Supplementary Table S1. Subcellular localization of the *B. napus* CYPs predicted by LocTree3.**

| **Name** | **Score** | **Expected Accuracy [%]** | **Localization Class** | **Gene Ontology Terms** | **Annotation Type** |
| --- | --- | --- | --- | --- | --- |
| BnCYP5 | 53 | 91 | cytoplasm | cytoplasm GO:0005737(IEA) | PSI-BLAST |
| BnCYP7-1 | 12 | 80 | secreted | extracellular region GO:0005576 | LOCTREE2 |
| BnCYP7-2 | 43 | 89 | cytoplasm | cytoplasm GO:0005737(IEA) | PSI-BLAST |
| BnCYP7-3 | 44 | 89 | secreted | extracellular region GO:0005576(IEA) | PSI-BLAST |
| BnCYP8 | 51 | 90 | nucleus | catalytic step 2 spliceosome GO:0071013(IDA) | PSI-BLAST |
| BnCYP10-1 | 33 | 86 | secreted | extracellular region GO:0005576 | LOCTREE2 |
| BnCYP10-2 | 40 | 88 | nucleus | mitotic spindle pole body GO:0044732(IDA); nuclear chromatin GO:0000790(IDA) | PSI-BLAST |
| BnCYP12-1 | 50 | 90 | cytoplasm | cytoplasm GO:0005737(IDA) | PSI-BLAST |
| BnCYP12-2 | 50 | 90 | nucleus | mitotic spindle pole body GO:0044732(IDA); nuclear chromatin GO:0000790(IDA) | PSI-BLAST |
| BnCYP13 | 50 | 90 | nucleus | nuclear matrix GO:0016363(IDA); nuclear speck GO:0016607(IEA) | PSI-BLAST |
| BnCYP14-1 | 51 | 90 | cytoplasm | cytoplasm GO:0005737(IEA) | PSI-BLAST |
| BnCYP14-2 | 51 | 90 | cytoplasm | cytoplasm GO:0005737(IEA) | PSI-BLAST |
| BnCYP14-3 | 49 | 90 | cytoplasm | cytoplasm GO:0005737(IEA) | PSI-BLAST |
| BnCYP14-4 | 36 | 87 | cytoplasm | cytoplasm GO:0005737(IEA) | PSI-BLAST |
| BnCYP14-5 | 82 | 96 | chloroplast | chloroplast stroma GO:0009570(IEA) | PSI-BLAST |
| BnCYP14-6 | 53 | 91 | cytoplasm | cytoplasm GO:0005737(IEA) | PSI-BLAST |
| BnCYP16 | 33 | 86 | cytoplasm | cytoplasm GO:0005737(IEA) | PSI-BLAST |
| BnCYP17-1 | 66 | 94 | cytoplasm | cytoplasm GO:0005737(IEA) | PSI-BLAST |
| BnCYP18-1 | 40 | 88 | nucleus | Golgi lumen GO:0005796(TAS); nucleus GO:0005634(TAS); ubiquitin ligase complex GO:0000151(IEA) | PSI-BLAST |
| BnCYP18-2 | 80 | 96 | cytoplasm | cytoplasm GO:0005737(IEA) | PSI-BLAST |
| BnCYP18-3 | 80 | 96 | cytoplasm | cytoplasm GO:0005737(IEA) | PSI-BLAST |
| BnCYP18-4 | 80 | 96 | cytoplasm | cytoplasm GO:0005737(IEA) | PSI-BLAST |
| BnCYP18-5 | 97 | 98 | cytoplasm | cytoplasm GO:0005737(IEA) | PSI-BLAST |
| BnCYP18-6 | 77 | 96 | cytoplasm | cytoplasm GO:0005737(IEA) | PSI-BLAST |
| BnCYP18-7 | 78 | 96 | cytoplasm | cytoplasm GO:0005737(IEA) | PSI-BLAST |
| BnCYP18-8 | 98 | 98 | cytoplasm | cytoplasm GO:0005737(IEA) | PSI-BLAST |
| BnCYP19-1 | 72 | 95 | cytoplasm | cytoplasm GO:0005737(IEA) | PSI-BLAST |
| BnCYP19-2 | 72 | 95 | cytoplasm | cytoplasm GO:0005737(IEA) | PSI-BLAST |
| BnCYP20 | 50 | 90 | cytoplasm | cytoplasm GO:0005737(IEA) | PSI-BLAST |
| BnCYP21-1 | 43 | 89 | nucleus | Golgi lumen GO:0005796(TAS); nucleus GO:0005634(TAS); ubiquitin ligase complex GO:0000151(IEA) | PSI-BLAST |
| BnCYP21-2 | 51 | 90 | cytoplasm | cytoplasm GO:0005737(IEA) | PSI-BLAST |
| BnCYP21-3 | 64 | 94 | cytoplasm | cytoplasm GO:0005737(IEA) | PSI-BLAST |
| BnCYP21-4 | 64 | 94 | cytoplasm | cytoplasm GO:0005737(IEA) | PSI-BLAST |
| **Name** | **Score** | **Expected Accuracy [%]** | **Localization Class** | **Gene Ontology Terms** | **Annotation Type** |
| BnCYP21-5 | 64 | 94 | cytoplasm | cytoplasm GO:0005737(IEA) | PSI-BLAST |
| BnCYP21-6 | 63 | 93 | cytoplasm | cytoplasm GO:0005737(IEA) | PSI-BLAST |
| BnCYP21-7 | 51 | 90 | cytoplasm | cytoplasm GO:0005737(IEA) | PSI-BLAST |
| BnCYP21-8 | 51 | 90 | cytoplasm | cytoplasm GO:0005737(IEA) | PSI-BLAST |
| BnCYP21-9 | 62 | 93 | cytoplasm | cytoplasm GO:0005737(IEA) | PSI-BLAST |
| BnCYP22-1 | 77 | 96 | chloroplast | chloroplast stroma GO:0009570(IDA); chloroplast thylakoid lumen GO:0009543(IEA); chloroplast thylakoid membrane GO:0009535(IDA); thylakoid lumen GO:0031977(IDA) | PSI-BLAST |
| BnCYP22-2 | 53 | 91 | cytoplasm | cytoplasm GO:0005737(IEA) | PSI-BLAST |
| BnCYP22-3 | 53 | 91 | cytoplasm | cytoplasm GO:0005737(IEA) | PSI-BLAST |
| BnCYP24-1 | 59 | 93 | cytoplasm | cytoplasm GO:0005737(IDA) | PSI-BLAST |
| BnCYP24-2 | 59 | 93 | cytoplasm | cytoplasm GO:0005737(IDA) | PSI-BLAST |
| BnCYP24-3 | 78 | 96 | chloroplast | chloroplast stroma GO:0009570(IEA) | PSI-BLAST |
| BnCYP24-4 | 46 | 89 | cytoplasm | cytoplasm GO:0005737(IEA) | PSI-BLAST |
| BnCYP24-5 | 48 | 89 | mitochondrion | mitochondrial matrix GO:0005759(IEA); mitochondrion GO:0005739(IDA) | PSI-BLAST |
| BnCYP24-6 | 49 | 90 | mitochondrion | mitochondrial matrix GO:0005759(IEA); mitochondrion GO:0005739(IDA) | PSI-BLAST |
| BnCYP24-7 | 46 | 89 | cytoplasm | cytoplasm GO:0005737(IEA) | PSI-BLAST |
| BnCYP25-1 | 16 | 82 | chloroplast | chloroplast thylakoid lumen GO:0009543(IEA) | PSI-BLAST |
| BnCYP25-2 | 17 | 82 | nucleus | nucleus GO:0005634(IDA) | PSI-BLAST |
| BnCYP25-3 | 22 | 84 | cytoplasm | cytosol GO:0005829(IDA); nucleus GO:0005634(IDA) | PSI-BLAST |
| BnCYP25-4 | 14 | 81 | chloroplast | chloroplast stroma GO:0009570(IEA) | PSI-BLAST |
| BnCYP25-5 | 13 | 81 | cytoplasm | cytoplasm GO:0005737(TAS) | PSI-BLAST |
| BnCYP26-1 | 16 | 82 | mitochondrion | mitochondrion GO:0005739(IEA) | PSI-BLAST |
| BnCYP26-2 | 83 | 96 | secreted | extracellular region GO:0005576 | LOCTREE2 |
| BnCYP26-3 | 15 | 81 | mitochondrion | mitochondrion GO:0005739(IEA) | PSI-BLAST |
| BnCYP26-4 | 57 | 92 | cytoplasm | cytoplasm GO:0005737(IEA) | PSI-BLAST |
| BnCYP26-5 | 16 | 82 | mitochondrion | mitochondrion GO:0005739(IEA) | PSI-BLAST |
| BnCYP27-1 | 43 | 89 | cytoplasm | cytoplasm GO:0005737(IEA) | PSI-BLAST |
| BnCYP27-2 | 92 | 97 | chloroplast | chloroplast stroma GO:0009570(IEA) | PSI-BLAST |
| BnCYP27-3 | 64 | 94 | cytoplasm | cytoplasm GO:0005737(IEA) | PSI-BLAST |
| BnCYP27-4 | 90 | 97 | chloroplast | chloroplast stroma GO:0009570(IEA) | PSI-BLAST |
| BnCYP27-5 | 64 | 94 | cytoplasm | cytoplasm GO:0005737(IEA) | PSI-BLAST |
| BnCYP28-1 | 87 | 97 | chloroplast | chloroplast stroma GO:0009570(IEA) | PSI-BLAST |
| BnCYP28-2 | 85 | 96 | chloroplast | chloroplast stroma GO:0009570(IEA) | PSI-BLAST |

| **Name** | **Score** | **Expected Accuracy [%]** | **Localization Class** | **Gene Ontology Terms** | **Annotation Type** |
| --- | --- | --- | --- | --- | --- |
| BnCYP30-1 | 20 | 83 | cytoplasm | cytosol GO:0005829(IDA); nucleus GO:0005634(IDA); spindle pole body GO:0005816(IEA) | PSI-BLAST |
| BnCYP30-2 | 20 | 83 | cytoplasm | cytosol GO:0005829(IDA); nucleus GO:0005634(IDA); spindle pole body GO:0005816(IEA) | PSI-BLAST |
| BnCYP31 | 72 | 95 | chloroplast | chloroplast stroma GO:0009570(IDA); chloroplast thylakoid lumen GO:0009543(IEA); chloroplast thylakoid membrane GO:0009535(IDA); thylakoid lumen GO:0031977(IDA) | PSI-BLAST |
| BnCYP34-1 | 17 | 82 | cytoplasm | cytoplasm GO:0005737(IEA) | PSI-BLAST |
| BnCYP34-2 | 15 | 81 | cytoplasm | cytosol GO:0005829(IDA); nucleus GO:0005634(IDA) | PSI-BLAST |
| BnCYP37-1 | 81 | 96 | secreted | extracellular region GO:0005576 | LOCTREE2 |
| BnCYP37-2 | 85 | 96 | secreted | extracellular region GO:0005576 | LOCTREE2 |
| BnCYP40-1 | 56 | 92 | cytoplasm | cytoplasm GO:0005737(IEA) | PSI-BLAST |
| BnCYP40-2 | 57 | 92 | cytoplasm | cytoplasm GO:0005737(IEA) | PSI-BLAST |
| BnCYP47-1 | 76 | 96 | secreted | extracellular region GO:0005576 | LOCTREE2 |
| BnCYP47-2 | 85 | 96 | chloroplast | chloroplast stroma GO:0009570(IDA); chloroplast thylakoid lumen GO:0009543(IEA); chloroplast thylakoid membrane GO:0009535(IDA); thylakoid lumen GO:0031977(IDA) | PSI-BLAST |
| BnCYP47-3 | 83 | 96 | chloroplast | chloroplast stroma GO:0009570(IDA); chloroplast thylakoid lumen GO:0009543(IEA); chloroplast thylakoid membrane GO:0009535(IDA); thylakoid lumen GO:0031977(IDA) | PSI-BLAST |
| BnCYP49 | 86 | 97 | chloroplast | chloroplast thylakoid lumen GO:0009543(IEA); chloroplast thylakoid membrane GO:0009535(IDA); thylakoid lumen GO:0031977(IDA) | PSI-BLAST |
| BnCYP50 | 86 | 97 | chloroplast | chloroplast thylakoid lumen GO:0009543(IEA); chloroplast thylakoid membrane GO:0009535(IDA); thylakoid lumen GO:0031977(IDA) | PSI-BLAST |
| BnCYP52 | 39 | 88 | nucleus | nuclear chromatin GO:0000790(ISS) | PSI-BLAST |
| BnCYP55 | 39 | 88 | nucleus | nuclear chromatin GO:0000790(ISS) | PSI-BLAST |
| BnCYP62 | 59 | 93 | cytoplasm | cytoplasm GO:0005737(IEA) | PSI-BLAST |
| BnCYP65-1 | 36 | 87 | nucleus | Golgi lumen GO:0005796(TAS); nucleus GO:0005634(TAS); ubiquitin ligase complex GO:0000151(IEA) | PSI-BLAST |
| **Name** | **Score** | **Expected Accuracy [%]** | **Localization Class** | **Gene Ontology Terms** | **Annotation Type** |
| BnCYP65-2 | 37 | 88 | mitochondrion | mitochondrion GO:0005739(IEA) | PSI-BLAST |
| BnCYP67 | 60 | 93 | cytoplasm | cytoplasm GO:0005737(IEA) | PSI-BLAST |
| BnCYP70-1 | 44 | 89 | nucleus | catalytic step 2 spliceosome GO:0071013(IDA) | PSI-BLAST |
| BnCYP70-2 | 44 | 89 | nucleus | catalytic step 2 spliceosome GO:0071013(IDA) | PSI-BLAST |
| BnCYP86-1 | 39 | 88 | cytoplasm | cytoplasm GO:0005737(IEA) | PSI-BLAST |
| BnCYP86-2 | 41 | 89 | mitochondrion | mitochondrial matrix GO:0005759(IEA) | PSI-BLAST |
| BnCYP112 | 19 | 83 | cytoplasm | cytoplasm GO:0005737(IEA); cytoskeleton GO:0005856(IEA) | PSI-BLAST |
| BnCYP146 | 52 | 90 | nucleus | mRNA cleavage and polyadenylation specificity factor complex GO:0005847(IEA); nucleus GO:0005634(IDA) | PSI-BLAST |

Supplementary Table S2. Experimentally verified *B. napus* CPYs by RNA-Seq. Only BnCYP mRNA reads that were found in all three samples were evaluated as being present under standard conditions and corresponding transcripts were therefore marked as identified in Table 3. BnCYPs that excluded due to this cut-off are highlighted (grey background).

| **Name** | **Gene name** | **Normalized counts**  **Sample 1** | **Normalized counts**  **Sample 2** | **Normalized counts**  **Sample 3** | **Mean** | **Standard deviation** |
| --- | --- | --- | --- | --- | --- | --- |
| BnCYP5 | BnaA01g25420D | 0 | 0 | 0 | 0 | 0 |
| BnCYP7-1 | BnaA09g37110D | 0 | 0 | 0 | 0 | 0 |
| BnCYP7-2 | BnaA01g36790D | 0 | 5 | 0 | 2 | 2 |
| BnCYP7-3 | BnaA01g36800D | 2 | 0 | 0 | 1 | 1 |
| BnCYP8 | BnaCnng42430D | 51 | 64 | 98 | 71 | 20 |
| BnCYP10-1 | BnaC02g14160D | 0 | 0 | 0 | 0 | 0 |
| BnCYP10-2 | BnaC06g10280D | 0 | 0 | 0 | 0 | 0 |
| BnCYP12-1 | BnaC02g10590D | 0 | 0 | 0 | 0 | 0 |
| BnCYP12-2 | BnaAnng41240D | 19 | 6 | 15 | 13 | 5 |
| BnCYP13 | BnaA02g07550D | 577 | 473 | 508 | 520 | 43 |
| BnCYP14-1 | BnaC04g41450D | 33 | 29 | 30 | 31 | 2 |
| BnCYP14-2 | BnaC04g41430D | 49 | 38 | 36 | 41 | 6 |
| BnCYP14-3 | BnaC04g41440D | 38 | 62 | 31 | 44 | 13 |
| BnCYP14-4 | BnaC08g28870D | 0 | 0 | 0 | 0 | 0 |
| BnCYP14-5 | BnaC09g34640D | 0 | 0 | 4 | 1 | 2 |
| BnCYP14-6 | BnaA01g25470D | 0 | 0 | 0 | 0 | 0 |
| BnCYP16 | BnaA02g10200D | 0 | 1 | 0 | 0 | 0 |
| BnCYP17-1 | BnaA01g25460D | 0 | 0 | 0 | 0 | 0 |
| BnCYP18-1 | BnaA05g08140D | 1651 | 1772 | 1788 | 1737 | 61 |
| BnCYP18-1 | BnaC04g09170D | 2122 | 2204 | 2441 | 2256 | 135 |
| BnCYP18-2 | BnaC01g03590D | 7478 | 6455 | 9222 | 7718 | 1142 |
| BnCYP18-3 | BnaA01g02340D | 504 | 307 | 515 | 442 | 96 |
| BnCYP18-4 | BnaC03g60160D | 1591 | 1451 | 1749 | 1597 | 122 |
| BnCYP18-4 | BnaA08g16920D | 9294 | 8584 | 11312 | 9730 | 1156 |
| BnCYP18-5 | BnaA09g08780D | 100801 | 79534 | 108256 | 96197 | 12169 |
| BnCYP18-6 | BnaA06g37360D | 28617 | 21454 | 26209 | 25427 | 2976 |
| BnCYP18-7 | BnaC07g47630D | 62676 | 58045 | 66402 | 62375 | 3418 |
| BnCYP18-8 | BnaC09g09060D | 53812 | 47761 | 63409 | 54994 | 6443 |
| BnCYP19-1 | BnaA09g35540D | 579 | 504 | 531 | 538 | 31 |
| BnCYP19-2 | BnaC08g26990D | 871 | 747 | 756 | 791 | 56 |
| BnCYP20 | BnaA01g36700D | 0 | 0 | 0 | 0 | 0 |
| BnCYP21-1 | BnaCnng08980D | 1401 | 1344 | 1369 | 1371 | 23 |
| BnCYP21-2 | BnaC04g06640D | 450 | 334 | 416 | 400 | 49 |
| BnCYP21-3 | BnaAnng15590D | 3893 | 4073 | 4670 | 4212 | 332 |
| BnCYP21-4 | BnaC03g12390D | 243 | 177 | 233 | 218 | 29 |

| **Name** | **Gene name** | **Normalized counts**  **Sample 1** | **Normalized counts**  **Sample 2** | **Normalized counts**  **Sample 3** | **Mean** | **Standard deviation** |
| --- | --- | --- | --- | --- | --- | --- |
| BnCYP21-5 | BnaC04g54560D | 4438 | 5076 | 5396 | 4970 | 398 |
| BnCYP21-6 | BnaAnng17350D | 300 | 218 | 342 | 287 | 51 |
| BnCYP21-7 | BnaA05g06380D | 411 | 470 | 448 | 443 | 24 |
| BnCYP21-8 | BnaC04g45890D | 338 | 398 | 367 | 368 | 25 |
| BnCYP21-9 | BnaA10g29520D | 1407 | 1174 | 1247 | 1276 | 97 |
| BnCYP21-9 | BnaC09g34020D | 1648 | 1650 | 1894 | 1731 | 115 |
| BnCYP22-1 | BnaCnng32070D | 2 | 0 | 2 | 1 | 1 |
| BnCYP22-2 | BnaA04g22160D | 358 | 340 | 457 | 385 | 52 |
| BnCYP22-3 | BnaC04g41460D | 65 | 61 | 46 | 57 | 8 |
| BnCYP24-1 | BnaC08g26840D | 720 | 807 | 721 | 750 | 41 |
| BnCYP24-2 | BnaA09g35470D | 660 | 623 | 759 | 681 | 57 |
| BnCYP24-3 | BnaA10g12350D | 0 | 0 | 0 | 0 | 0 |
| BnCYP24-4 | BnaA08g10930D | 392 | 332 | 442 | 389 | 45 |
| BnCYP24-5 | BnaC01g03530D | 877 | 906 | 846 | 876 | 25 |
| BnCYP24-6 | BnaA01g02260D | 958 | 1110 | 941 | 1003 | 76 |
| BnCYP24-7 | BnaC03g65620D | 10 | 9 | 13 | 11 | 2 |
| BnCYP25-1 | BnaA08g20010D | 406 | 488 | 512 | 469 | 45 |
| BnCYP25-2 | BnaC08g48540D | 352 | 407 | 361 | 373 | 24 |
| BnCYP25-3 | BnaA09g29400D | 195 | 208 | 226 | 210 | 13 |
| BnCYP25-4 | BnaA03g21710D | 5515 | 6018 | 5840 | 5791 | 208 |
| BnCYP25-5 | BnaC03g25990D | 5120 | 5997 | 5777 | 5631 | 372 |
| BnCYP26-1 | BnaA05g30740D | 1117 | 1397 | 1362 | 1292 | 125 |
| BnCYP26-2 | BnaA08g08200D | 173 | 118 | 180 | 157 | 28 |
| BnCYP26-3 | BnaC05g45190D | 912 | 959 | 995 | 955 | 34 |
| BnCYP26-4 | BnaA04g17840D | 14 | 30 | 60 | 35 | 19 |
| BnCYP26-5 | BnaCnng32180D | 2606 | 3147 | 2944 | 2899 | 223 |
| BnCYP27-1 | BnaC05g44950D | 0 | 0 | 0 | 0 | 0 |
| BnCYP27-2 | BnaC08g31970D | 32296 | 29980 | 37656 | 33311 | 3215 |
| BnCYP27-3 | BnaC03g05790D | 7566 | 6238 | 7335 | 7047 | 579 |
| BnCYP27-4 | BnaA09g39610D | 21083 | 19180 | 23644 | 21302 | 1829 |
| BnCYP27-5 | BnaA03g04200D | 1900 | 1472 | 1708 | 1693 | 175 |
| BnCYP28-1 | BnaA04g27460D | 1134 | 887 | 1120 | 1047 | 114 |
| BnCYP28-2 | BnaC04g21530D | 3544 | 3653 | 3453 | 3550 | 82 |
| BnCYP30-1 | BnaC08g08050D | 2486 | 2625 | 2430 | 2514 | 82 |
| BnCYP30-2 | BnaA08g07170D | 1558 | 1516 | 1791 | 1621 | 121 |
| BnCYP31 | BnaC07g00280D | 0 | 0 | 0 | 0 | 0 |
| BnCYP34-1 | BnaC02g22620D | 1050 | 757 | 1057 | 955 | 140 |
| BnCYP34-2 | BnaA02g16680D | 482 | 264 | 380 | 376 | 89 |
| BnCYP37-1 | BnaC01g21230D | 1677 | 1635 | 1814 | 1709 | 76 |
| BnCYP37-2 | BnaA01g17950D | 563 | 523 | 669 | 585 | 61 |
| BnCYP40-1 | BnaC07g05260D | 303 | 242 | 234 | 259 | 31 |
| **Name** | **Gene name** | **Normalized counts**  **Sample 1** | **Normalized counts**  **Sample 2** | **Normalized counts**  **Sample 3** | **Mean** | **Standard deviation** |
| BnCYP40-2 | BnaA07g04040D | 465 | 404 | 389 | 419 | 33 |
| BnCYP47-1 | BnaC08g10870D | 143 | 232 | 164 | 179 | 38 |
| BnCYP47-2 | BnaA05g33830D | 4729 | 3389 | 4406 | 4174 | 571 |
| BnCYP47-3 | BnaC05g48850D | 888 | 618 | 843 | 783 | 118 |
| BnCYP49 | BnaA05g24020D | 1672 | 1728 | 1637 | 1679 | 38 |
| BnCYP50 | BnaC05g38110D | 1391 | 1363 | 1219 | 1324 | 75 |
| BnCYP52 | BnaC01g05170D | 227 | 260 | 273 | 253 | 19 |
| BnCYP55 | BnaA01g03810D | 63 | 125 | 105 | 98 | 26 |
| BnCYP62 | BnaAnng12550D | 319 | 368 | 341 | 343 | 20 |
| BnCYP65-1 | BnaA06g24990D | 308 | 393 | 291 | 331 | 45 |
| BnCYP65-2 | BnaC03g48580D | 379 | 386 | 311 | 359 | 34 |
| BnCYP67 | BnaC04g20680D | 684 | 577 | 580 | 614 | 50 |
| BnCYP70-1 | BnaC03g54740D | 776 | 934 | 696 | 802 | 99 |
| BnCYP70-2 | BnaA06g18830D | 803 | 986 | 736 | 841 | 106 |
| BnCYP86-1 | BnaA01g04590D | 78 | 73 | 83 | 78 | 4 |
| BnCYP86-2 | BnaC01g06080D | 41 | 53 | 44 | 46 | 5 |
| BnCYP112 | BnaC03g71020D | 512 | 458 | 438 | 469 | 31 |
| BnCYP146 | BnaA03g29520D | 1396 | 1717 | 1763 | 1625 | 163 |

Supplementary **Table S3.** BnCYPs identified by LC-MS/MS from denatured leaf extracts (n = 3).

| **Accession** | **Peptide count** | **Unique peptides** | **Confidence score** | **Average Intensity** | **Standard deviation** |
| --- | --- | --- | --- | --- | --- |
| BnCYP18-4 | 3 | 1 | 296,47 | 337377 | 105561 |
| BnCYP18-5 | 7 | 5 | 465,66 | 43503992 | 7779564 |
| BnCYP19-2 | 4 | 1 | 115,72 | 10982 | 19021 |
| BnCYP21-3 | 6 | 2 | 243,16 | 3141689 | 244704 |
| BnCYP24-1 | 3 | 1 | 79,24 | 24111638 | 6333588 |
| BnCYP27-2 | 15 | 2 | 1136,96 | 2881346 | 3702201 |
| BnCYP27-3 | 3 | 3 | 114,15 | 2904360 | 181212 |
| BnCYP27-4 | 14 | 5 | 1014,07 | 55211602 | 13190532 |
| BnCYP28-1 | 7 | 1 | 686,22 | 243896 | 400570 |
| BnCYP30-1 | 4 | 4 | 163,49 | 4716821 | 2010199 |
| BnCYP34-1 | 3 | 3 | 135,86 | 1050959 | 296099 |
| BnCYP47-2 | 6 | 1 | 340,22 | 539535 | 175077 |
| BnCYP49 | 3 | 1 | 222,44 | 414504 | 85817 |
| BnCYP62 | 2 | 1 | 50,74 | 70635 | 24032 |

**Supplementary Table S4. BnCYPs identified by LC-MS/MS from denatured phloem samples (n = 4).**

| **Accession** | **Peptide count** | **Unique peptides** | **Confidence score** | **Average Intensity** | **Standard deviation** |
| --- | --- | --- | --- | --- | --- |
| BnCYP13 | 4 | 1 | 80,89 | 1171909 | 44074 |
| BnCYP18-1 | 14 | 11 | 583,77 | 10349599 | 3401379 |
| BnCYP18-2 | 16 | 2 | 924,25 | 270353 | 83307 |
| BnCYP18-4 | 17 | 5 | 1277,69 | 6124435 | 866598 |
| BnCYP18-5 | 24 | 17 | 2234,23 | 628223562 | 117532797 |
| BnCYP18-6 | 18 | 2 | 1505,8 | 20790145 | 4257558 |
| BnCYP18-7 | 17 | 1 | 1415,43 | 17245155 | 4125944 |
| BnCYP19-1 | 12 | 4 | 299,61 | 19609582 | 3466795 |
| BnCYP19-2 | 9 | 1 | 223,52 | 10165074 | 2587599 |
| BnCYP21-1 | 6 | 6 | 208,55 | 3285155 | 305100 |
| BnCYP21-3 | 13 | 6 | 334,1 | 3830250 | 1133635 |
| BnCYP21-4 | 13 | 2 | 309,54 | 2109157 | 365989 |
| BnCYP21-9 | 12 | 2 | 232,37 | 312213 | 69406 |
| BnCYP22-2 | 11 | 1 | 353,8 | 709335 | 34186 |
| BnCYP27-2 | 13 | 2 | 462,82 | 2378814 | 690082 |
| BnCYP27-3 | 7 | 6 | 70,01 | 7869880 | 4366559 |
| BnCYP27-4 | 14 | 6 | 440,71 | 9040123 | 611432 |
| BnCYP28-1 | 13 | 3 | 393,55 | 813148 | 217092 |
| BnCYP28-2 | 12 | 1 | 425,84 | 55655 | 15288 |
| BnCYP86-2 | 18 | 16 | 171,2 | 16825858 | 2954867 |

Supplementary **Table S5.** Peptides mapped to identified BnCYPs from leaf extracts. Unique peptides, used for quantification, are marked with an *.

| **Accession** | **Retention time (min)** | **m/z** | **Charge** | **Measured mass (Da)** | **Mass error (ppm)** | **Sequence** | **Modifications** |
| --- | --- | --- | --- | --- | --- | --- | --- |
| BnCYP18-4 | 37.70 | 417.70 | 2 | 833.39 | 1.70 | TDWLDGK |  |
| BnCYP18-4 | 53.74 | 542.31 | 3 | 1623.90 | 3.64 | HVVFGQVVEGLDVVK |  |
| BnCYP18-4 | 53.77 | 812.96 | 2 | 1623.90 | 2.46 | HVVFGQVVEGLDVVK |  |
| BnCYP18-4 | 23.08 | 385.85 | 3 | 1154.54 | 0.84 | FADENFERK* |  |
| BnCYP18-4 | 23.11 | 578.28 | 2 | 1154.54 | 1.97 | FADENFERK* |  |
| BnCYP18-5 | 13.98 | 371.22 | 3 | 1110.63 | 2.30 | GSAFHRVIPK* |  |
| BnCYP18-5 | 13.98 | 556.32 | 2 | 1110.63 | 1.81 | GSAFHRVIPK* |  |
| BnCYP18-5 | 32.96 | 403.71 | 2 | 805.40 | 2.01 | TSWLDGK* |  |
| BnCYP18-5 | 49.69 | 587.78 | 2 | 1173.54 | 3.36 | VYFDMTVGDK* |  |
| BnCYP18-5 | 23.08 | 513.77 | 2 | 1025.52 | 2.23 | FKDENFVK |  |
| BnCYP18-5 | 23.05 | 342.85 | 3 | 1025.52 | 1.54 | FKDENFVK |  |
| BnCYP18-5 | 55.72 | 719.38 | 3 | 2155.12 | 2.62 | HVVFGQVVEGMDVVRDIEK* |  |
| BnCYP18-5 | 55.66 | 1078.57 | 2 | 2155.12 | 3.43 | HVVFGQVVEGMDVVRDIEK* |  |
| BnCYP18-5 | 58.91 | 1049.52 | 2 | 2097.02 | 3.62 | IVMELYADTVPETAENFR* |  |
| BnCYP18-5 | 58.91 | 700.01 | 3 | 2097.01 | 2.64 | IVMELYADTVPETAENFR* |  |
| BnCYP18-5 | 10.21 | 440.23 | 2 | 878.45 | 1.25 | DENFVKK |  |
| BnCYP19-2 | 8.41 | 343.70 | 2 | 685.39 | 0.70 | HVVFGK |  |
| BnCYP19-2 | 30.26 | 496.78 | 2 | 991.54 | 3.17 | VVDGYNVVK |  |
| BnCYP19-2 | 62.89 | 526.31 | 2 | 1050.61 | 2.86 | VFFDILIGK |  |
| BnCYP19-2 | 60.64 | 431.60 | 3 | 1291.79 | 2.07 | VFFDILIGKLK* |  |
| BnCYP21-3 | 8.41 | 343.70 | 2 | 685.39 | 0.70 | HVVFGK |  |
| BnCYP21-3 | 39.05 | 569.30 | 2 | 1136.59 | 3.27 | VVQGMDVVYK* |  |
| BnCYP21-3 | 26.96 | 435.70 | 2 | 869.39 | 1.50 | FADENFK |  |
| BnCYP21-3 | 7.64 | 373.87 | 3 | 1118.58 | 8.31 | ALCTGEKGVGK | [3] Carbamido-methyl (C) |
| BnCYP21-3 | 7.68 | 560.30 | 2 | 1118.58 | 8.39 | ALCTGEKGVGK | [3] Carbamido-methyl (C) |
| BnCYP21-3 | 35.33 | 371.20 | 3 | 1110.57 | 1.03 | FADENFKLK |  |
| BnCYP21-3 | 53.88 | 821.15 | 4 | 3280.57 | 5.15 | FHRIIPSFMIQGGDFTHGNGMGGESIYGQK* |  |
| BnCYP24-1 | 24.86 | 335.85 | 3 | 1004.54 | 8.43 | VLSGMEVVR* | [5] Oxidation (M) |
| BnCYP24-1 | 24.86 | 503.28 | 2 | 1004.54 | 9.49 | VLSGMEVVR* | [5] Oxidation (M) |
| BnCYP24-1 | 26.96 | 435.70 | 2 | 869.39 | 1.50 | FADENFK |  |
| BnCYP24-1 | 35.33 | 371.20 | 3 | 1110.57 | 1.03 | FADENFKLK |  |
| BnCYP27-2 | 10.37 | 348.87 | 3 | 1043.59 | 2.18 | GSSFHRIIK |  |
| BnCYP27-2 | 10.39 | 522.80 | 2 | 1043.59 | 1.85 | GSSFHRIIK |  |
| BnCYP27-2 | 59.42 | 637.86 | 2 | 1273.71 | 3.16 | IVMGLFGDVVPK |  |
| BnCYP27-2 | 45.71 | 571.77 | 2 | 1141.53 | 3.54 | FEDENFTLK |  |
| BnCYP27-2 | 45.13 | 571.77 | 2 | 1141.53 | 4.06 | FEDENFTLK |  |
| BnCYP27-2 | 46.41 | 672.36 | 2 | 1342.71 | 1.15 | HVVFGQVIEGMK |  |
| BnCYP27-2 | 46.41 | 448.58 | 3 | 1342.71 | 1.99 | HVVFGQVIEGMK |  |
| BnCYP27-2 | 26.56 | 338.69 | 2 | 675.36 | 1.58 | AFDVPK |  |
| BnCYP27-2 | 39.47 | 540.95 | 3 | 1619.82 | 2.56 | TLESQETRAFDVPK |  |
| **Accession** | **Retention time (min)** | **m/z** | **Charge** | **Measured mass (Da)** | **Mass error (ppm)** | **Sequence** | **Modifications** |
| BnCYP27-2 | 39.50 | 810.92 | 2 | 1619.82 | 1.89 | TLESQETRAFDVPK |  |
| BnCYP27-2 | 15.69 | 383.20 | 2 | 764.38 | 0.30 | TVENFR |  |
| BnCYP27-2 | 32.67 | 437.23 | 2 | 872.44 | 2.23 | TPWLDNK* |  |
| BnCYP27-2 | 54.78 | 645.86 | 2 | 1289.71 | 2.01 | IVMGLFGDVVPK | [3] Oxidation (M) |
| BnCYP27-2 | 19.26 | 444.58 | 3 | 1330.72 | 0.92 | LVRTLESQETR |  |
| BnCYP27-2 | 19.29 | 666.37 | 2 | 1330.72 | 0.64 | LVRTLESQETR |  |
| BnCYP27-2 | 19.52 | 666.37 | 2 | 1330.72 | 0.60 | LVRTLESQETR |  |
| BnCYP27-2 | 37.75 | 518.26 | 3 | 1551.77 | -2.79 | TVENFRVLCTGEK | [9] Carbamido-methyl (C) |
| BnCYP27-2 | 15.11 | 402.74 | 2 | 803.46 | 2.05 | AFDVPKK |  |
| BnCYP27-2 | 61.00 | 1218.07 | 2 | 2434.13 | 6.27 | DFMIQGGDFTEGNGTGGISIYGAK |  |
| BnCYP27-2 | 61.00 | 812.38 | 3 | 2434.12 | 4.11 | DFMIQGGDFTEGNGTGGISIYGAK |  |
| BnCYP27-2 | 36.84 | 680.36 | 2 | 1358.70 | 1.25 | HVVFGQVIEGMK | [11] Oxidation (M) |
| BnCYP27-2 | 46.41 | 453.91 | 3 | 1358.70 | 1.96 | HVVFGQVIEGMK | [11] Oxidation (M) |
| BnCYP27-2 | 59.91 | 919.21 | 4 | 3672.80 | 4.36 | HTGPGILSMANAGPNTNGSQFFICTVKTPWLDNK* | [24] Carbamido-methyl (C) |
| BnCYP27-3 | 54.91 | 697.87 | 2 | 1393.73 | 1.49 | VYFDISVGNPVGK* |  |
| BnCYP27-3 | 16.54 | 319.84 | 3 | 956.51 | 2.11 | SVYGRTFK* |  |
| BnCYP27-3 | 17.18 | 571.96 | 3 | 1712.85 | 2.86 | LIEEQETDRGDRPR* |  |
| BnCYP27-4 | 32.64 | 513.81 | 2 | 1025.60 | 2.19 | HVVFGQVIK* |  |
| BnCYP27-4 | 32.59 | 342.88 | 3 | 1025.60 | 1.78 | HVVFGQVIK* |  |
| BnCYP27-4 | 10.37 | 348.87 | 3 | 1043.59 | 2.18 | GSSFHRIIK |  |
| BnCYP27-4 | 10.39 | 522.80 | 2 | 1043.59 | 1.85 | GSSFHRIIK |  |
| BnCYP27-4 | 45.71 | 571.77 | 2 | 1141.53 | 3.54 | FEDENFTLK |  |
| BnCYP27-4 | 45.13 | 571.77 | 2 | 1141.53 | 4.06 | FEDENFTLK |  |
| BnCYP27-4 | 26.56 | 338.69 | 2 | 675.36 | 1.58 | AFDVPK |  |
| BnCYP27-4 | 34.94 | 408.72 | 2 | 815.42 | 1.86 | TPWLDGK* |  |
| BnCYP27-4 | 39.47 | 540.95 | 3 | 1619.82 | 2.56 | TLESQETRAFDVPK |  |
| BnCYP27-4 | 39.50 | 810.92 | 2 | 1619.82 | 1.89 | TLESQETRAFDVPK |  |
| BnCYP27-4 | 15.69 | 383.20 | 2 | 764.38 | 0.30 | TVENFR |  |
| BnCYP27-4 | 19.26 | 444.58 | 3 | 1330.72 | 0.92 | LVRTLESQETR |  |
| BnCYP27-4 | 19.29 | 666.37 | 2 | 1330.72 | 0.64 | LVRTLESQETR |  |
| BnCYP27-4 | 19.52 | 666.37 | 2 | 1330.72 | 0.60 | LVRTLESQETR |  |
| BnCYP27-4 | 35.02 | 448.26 | 3 | 1341.76 | 1.31 | HVVFGQVIKGMK* |  |
| BnCYP27-4 | 35.02 | 671.89 | 2 | 1341.76 | 0.65 | HVVFGQVIKGMK* |  |
| BnCYP27-4 | 58.97 | 652.85 | 2 | 1303.69 | 3.89 | IEMGLFGDVVPK* |  |
| BnCYP27-4 | 37.75 | 518.26 | 3 | 1551.77 | -2.79 | TVENFRVLCTGEK | [9] Carbamido-methyl (C) |
| BnCYP27-4 | 15.11 | 402.74 | 2 | 803.46 | 2.05 | AFDVPKK |  |
| BnCYP27-4 | 20.59 | 548.29 | 2 | 1094.57 | -1.22 | QFPGAYATIK* |  |
| BnCYP27-4 | 20.59 | 365.87 | 3 | 1094.57 | -0.96 | QFPGAYATIK* |  |
| BnCYP27-4 | 61.00 | 1218.07 | 2 | 2434.13 | 6.27 | DFMIQGGDFTEGNGTGGISIYGAK |  |
| BnCYP27-4 | 61.00 | 812.38 | 3 | 2434.12 | 4.11 | DFMIQGGDFTEGNGTGGISIYGAK |  |
| BnCYP28-1 | 10.37 | 348.87 | 3 | 1043.59 | 2.18 | GSSFHRIIK |  |
| BnCYP28-1 | 10.39 | 522.80 | 2 | 1043.59 | 1.85 | GSSFHRIIK |  |

| **Accession** | **Retention time (min)** | **m/z** | **Charge** | **Measured mass (Da)** | **Mass error (ppm)** | **Sequence** | **Modifications** |
| --- | --- | --- | --- | --- | --- | --- | --- |
| BnCYP28-1 | 45.71 | 571.77 | 2 | 1141.53 | 3.54 | FEDENFTLK |  |
| BnCYP28-1 | 45.13 | 571.77 | 2 | 1141.53 | 4.06 | FEDENFTLK |  |
| BnCYP28-1 | 46.41 | 672.36 | 2 | 1342.71 | 1.15 | HVVFGQVIEGMK |  |
| BnCYP28-1 | 46.41 | 448.58 | 3 | 1342.71 | 1.99 | HVVFGQVIEGMK |  |
| BnCYP28-1 | 15.69 | 383.20 | 2 | 764.38 | 0.30 | TVENFR |  |
| BnCYP28-1 | 61.00 | 1218.07 | 2 | 2434.13 | 6.27 | DFMIQGGDFTEGNGTGGISIYGAK |  |
| BnCYP28-1 | 61.00 | 812.38 | 3 | 2434.12 | 4.11 | DFMIQGGDFTEGNGTGGISIYGAK |  |
| BnCYP28-1 | 36.84 | 680.36 | 2 | 1358.70 | 1.25 | HVVFGQVIEGMK | [11] Oxidation (M) |
| BnCYP28-1 | 46.41 | 453.91 | 3 | 1358.70 | 1.96 | HVVFGQVIEGMK | [11] Oxidation (M) |
| BnCYP28-1 | 60.08 | 651.87 | 2 | 1301.72 | 1.33 | IVMGLFGDVVPR* |  |
| BnCYP30-1 | 43.74 | 563.33 | 2 | 1124.65 | 2.26 | VTKPSLSPSLP* |  |
| BnCYP30-1 | 36.65 | 375.89 | 3 | 1124.64 | 0.75 | VTKPSLSPSLP* |  |
| BnCYP30-1 | 36.65 | 563.33 | 2 | 1124.65 | 1.67 | VTKPSLSPSLP* |  |
| BnCYP30-1 | 32.28 | 338.53 | 3 | 1012.58 | 0.35 | SLWNRPLK* |  |
| BnCYP30-1 | 60.18 | 626.85 | 2 | 1251.68 | 3.39 | IFPGQFFLAGR* |  |
| BnCYP30-1 | 44.32 | 438.77 | 2 | 875.52 | 1.52 | VVLGLYGR* |  |
| BnCYP34-1 | 30.97 | 443.24 | 3 | 1326.69 | 0.96 | IMPGYVQHGGIR* |  |
| BnCYP34-1 | 24.45 | 472.24 | 3 | 1413.69 | 0.54 | TVRDNTSSPYFK* |  |
| BnCYP34-1 | 24.55 | 707.85 | 2 | 1413.69 | 0.47 | TVRDNTSSPYFK* |  |
| BnCYP34-1 | 25.09 | 412.73 | 2 | 823.45 | 8.71 | FSSIVSGK* |  |
| BnCYP47-2 | 45.56 | 570.29 | 2 | 1138.57 | 2.48 | SMIVAGFAESK |  |
| BnCYP47-2 | 6.95 | 370.87 | 3 | 1109.58 | 0.24 | KDHGNELIGK* |  |
| BnCYP47-2 | 6.92 | 555.80 | 2 | 1109.58 | 1.48 | KDHGNELIGK* |  |
| BnCYP47-2 | 57.48 | 625.98 | 3 | 1874.93 | 5.05 | LEAGMQDMLQIVEDRK |  |
| BnCYP47-2 | 36.03 | 578.29 | 2 | 1154.57 | 2.34 | SMIVAGFAESK | [2] Oxidation (M) |
| BnCYP47-2 | 5.93 | 439.23 | 2 | 876.45 | -0.07 | GRATVDMK |  |
| BnCYP47-2 | 39.99 | 648.30 | 2 | 1294.58 | 2.84 | HFYDGMEIQR |  |
| BnCYP47-2 | 39.99 | 432.53 | 3 | 1294.58 | 2.53 | HFYDGMEIQR |  |
| BnCYP49 | 23.00 | 556.29 | 2 | 1110.56 | 1.65 | LVTSGAYDGTK* |  |
| BnCYP49 | 37.70 | 823.42 | 2 | 1644.83 | 1.63 | LNTVNQAVITEDGSGK |  |
| BnCYP49 | 49.72 | 1028.54 | 2 | 2055.07 | 2.89 | LIEGQDRLILPAQNNSSST |  |
| BnCYP62 | 8.41 | 343.70 | 2 | 685.39 | 0.70 | HVVFGK |  |
| BnCYP62 | 6.90 | 464.76 | 2 | 927.50 | 1.44 | QPISQDLK* |  |

Supplementary **Table S6.** Peptides mapped to identified BnCYPs from phloem extracts. Unique peptides, used for quantification, are marked with an *.

| **Accession** | **Retention time (min)** | **m/z** | **Charge** | **Measured mass (Da)** | **Mass error (ppm)** | **Sequence** | **Modifications** |
| --- | --- | --- | --- | --- | --- | --- | --- |
| BnCYP13 | 15.94 | 444.76 | 2 | 887.51 | 1.48 | GGQSILSVK* |  |
| BnCYP13 | 38.27 | 432.73 | 2 | 863.45 | 1.69 | NFVELSR |  |
| BnCYP13 | 15.60 | 432.73 | 2 | 863.45 | 2.23 | NFVELSR |  |
| BnCYP13 | 29.24 | 510.78 | 2 | 1019.55 | 2.06 | NFVELSRR |  |
| BnCYP13 | 28.30 | 510.78 | 2 | 1019.55 | 0.88 | NFVELSRR |  |
| BnCYP13 | 28.35 | 340.86 | 3 | 1019.55 | -1.83 | NFVELSRR |  |
| BnCYP13 | 39.64 | 473.90 | 3 | 1418.68 | 3.60 | DFIVQGGDPTGTGR |  |
| BnCYP18-1 | 37.15 | 416.74 | 2 | 831.46 | -0.72 | GMEVIKR* |  |
| BnCYP18-1 | 38.27 | 432.73 | 2 | 863.45 | 1.69 | NFVELSR |  |
| BnCYP18-1 | 15.60 | 432.73 | 2 | 863.45 | 2.23 | NFVELSR |  |
| BnCYP18-1 | 42.60 | 480.58 | 3 | 1438.71 | 2.73 | RGYYDNVLFHR* |  |
| BnCYP18-1 | 42.60 | 720.36 | 2 | 1438.71 | 1.73 | RGYYDNVLFHR* |  |
| BnCYP18-1 | 50.35 | 642.31 | 2 | 1282.61 | 1.11 | GYYDNVLFHR* |  |
| BnCYP18-1 | 50.38 | 428.54 | 3 | 1282.61 | 1.54 | GYYDNVLFHR* |  |
| BnCYP18-1 | 39.08 | 416.56 | 3 | 1246.65 | 1.54 | FEDEIKPELK* |  |
| BnCYP18-1 | 18.70 | 365.70 | 2 | 729.39 | 1.91 | HTIFGR* |  |
| BnCYP18-1 | 29.18 | 319.00 | 6 | 1907.97 | 0.08 | LGSVQTDNTDRPIHEVK* |  |
| BnCYP18-1 | 23.53 | 319.00 | 6 | 1907.97 | -0.50 | LGSVQTDNTDRPIHEVK* |  |
| BnCYP18-1 | 27.38 | 637.00 | 3 | 1907.97 | 2.00 | LGSVQTDNTDRPIHEVK* |  |
| BnCYP18-1 | 65.42 | 1119.23 | 3 | 3354.67 | 0.36 | HTGAGILSMANAGPNTNGSQFFITLAPAPSLDGK* |  |
| BnCYP18-1 | 29.24 | 510.78 | 2 | 1019.55 | 2.06 | NFVELSRR |  |
| BnCYP18-1 | 28.30 | 510.78 | 2 | 1019.55 | 0.88 | NFVELSRR |  |
| BnCYP18-1 | 28.35 | 340.86 | 3 | 1019.55 | -1.83 | NFVELSRR |  |
| BnCYP18-1 | 23.98 | 517.03 | 4 | 2064.08 | 2.37 | RLGSVQTDNTDRPIHEVK* |  |
| BnCYP18-1 | 23.98 | 689.03 | 3 | 2064.08 | 2.40 | RLGSVQTDNTDRPIHEVK* |  |
| BnCYP18-1 | 39.64 | 473.90 | 3 | 1418.68 | 3.60 | DFIVQGGDPTGTGR |  |
| BnCYP18-1 | 26.91 | 364.53 | 3 | 1090.57 | 4.43 | VCRGMEVIK* | [2] Carbamido-methyl (C) |
| BnCYP18-1 | 28.41 | 382.71 | 6 | 2290.22 | -6.87 | LGSVQTDNTDRPIHEVKILR* |  |
| BnCYP18-1 | 20.50 | 338.69 | 2 | 675.36 | 1.86 | GMEVIK* |  |
| BnCYP18-2 | 34.64 | 495.27 | 2 | 988.53 | 2.78 | VVEGMEVVK |  |
| BnCYP18-2 | 4.83 | 330.51 | 3 | 988.52 | -8.05 | VVEGMEVVK |  |
| BnCYP18-2 | 60.32 | 748.87 | 2 | 1495.72 | 1.83 | VFFDMTLDGAPAGR |  |
| BnCYP18-2 | 60.38 | 499.58 | 3 | 1495.72 | 1.65 | VFFDMTLDGAPAGR |  |
| BnCYP18-2 | 52.33 | 714.69 | 3 | 2141.05 | 3.48 | IVMELYKDTTPNTAENFR |  |
| BnCYP18-2 | 52.36 | 1071.53 | 2 | 2141.05 | 2.30 | IVMELYKDTTPNTAENFR |  |
| BnCYP18-2 | 10.45 | 343.70 | 2 | 685.39 | 0.50 | HVVFGK |  |
| BnCYP18-2 | 6.20 | 413.75 | 2 | 825.49 | -0.63 | GKPLHFK |  |
| BnCYP18-2 | 7.22 | 413.75 | 2 | 825.49 | -0.07 | GKPLHFK |  |
| BnCYP18-2 | 46.38 | 448.25 | 2 | 894.49 | 1.96 | IVMELYK |  |
| BnCYP18-2 | 5.39 | 337.67 | 2 | 673.33 | -0.58 | GSAFHR |  |

| **Accession** | **Retention time (min)** | **m/z** | **Charge** | **Measured mass (Da)** | **Mass error (ppm)** | **Sequence** | **Modifications** |
| --- | --- | --- | --- | --- | --- | --- | --- |
| BnCYP18-2 | 4.81 | 337.67 | 2 | 673.33 | -0.62 | GSAFHR |  |
| BnCYP18-2 | 6.77 | 337.67 | 2 | 673.33 | -1.14 | GSAFHR |  |
| BnCYP18-2 | 5.76 | 337.67 | 2 | 673.33 | -1.11 | GSAFHR |  |
| BnCYP18-2 | 3.93 | 337.67 | 2 | 673.33 | 0.29 | GSAFHR |  |
| BnCYP18-2 | 3.05 | 337.67 | 2 | 673.33 | -0.06 | GSAFHR |  |
| BnCYP18-2 | 0.07 | 337.67 | 2 | 673.33 | 0.66 | GSAFHR |  |
| BnCYP18-2 | 31.79 | 633.29 | 2 | 1264.57 | 2.68 | DTTPNTAENFR |  |
| BnCYP18-2 | 32.42 | 633.29 | 2 | 1264.57 | 1.06 | DTTPNTAENFR |  |
| BnCYP18-2 | 5.33 | 318.87 | 3 | 953.58 | -0.32 | KGKPLHFK |  |
| BnCYP18-2 | 5.33 | 477.80 | 2 | 953.58 | -0.01 | KGKPLHFK |  |
| BnCYP18-2 | 4.70 | 477.80 | 2 | 953.58 | 1.40 | KGKPLHFK |  |
| BnCYP18-2 | 34.76 | 503.27 | 2 | 1004.52 | 2.17 | VVEGMEVVK | [5] Oxidation (M) |
| BnCYP18-2 | 20.50 | 503.27 | 2 | 1004.52 | 1.86 | VVEGMEVVK | [5] Oxidation (M) |
| BnCYP18-2 | 20.91 | 503.27 | 2 | 1004.52 | 1.47 | VVEGMEVVK | [5] Oxidation (M) |
| BnCYP18-2 | 22.13 | 476.26 | 2 | 950.51 | 5.33 | VGSSSGTTKK |  |
| BnCYP18-2 | 18.92 | 499.75 | 2 | 997.49 | 2.25 | FKDENFAK* |  |
| BnCYP18-2 | 18.92 | 333.50 | 3 | 997.49 | 1.49 | FKDENFAK* |  |
| BnCYP18-2 | 54.99 | 756.86 | 2 | 1511.71 | 0.76 | VFFDMTLDGAPAGR | [5] Oxidation (M) |
| BnCYP18-2 | 60.32 | 756.86 | 2 | 1511.71 | 3.23 | VFFDMTLDGAPAGR | [5] Oxidation (M) |
| BnCYP18-2 | 37.15 | 509.26 | 2 | 1016.50 | 3.73 | VVVADCGQVA | [6] Carbamido-methyl (C) |
| BnCYP18-2 | 7.02 | 389.69 | 2 | 777.37 | 0.37 | ALCTGEK | [3] Carbamido-methyl (C) |
| BnCYP18-2 | 12.60 | 362.17 | 2 | 722.32 | 0.86 | DENFAK* |  |
| BnCYP18-4 | 43.63 | 479.93 | 3 | 1436.76 | 2.49 | IVMELYTDKTPK |  |
| BnCYP18-4 | 43.63 | 719.39 | 2 | 1436.76 | 1.85 | IVMELYTDKTPK |  |
| BnCYP18-4 | 42.95 | 479.93 | 3 | 1436.77 | 5.57 | IVMELYTDKTPK |  |
| BnCYP18-4 | 56.23 | 542.31 | 3 | 1623.90 | 3.19 | HVVFGQVVEGLDVVK |  |
| BnCYP18-4 | 56.23 | 812.96 | 2 | 1623.90 | 2.25 | HVVFGQVVEGLDVVK |  |
| BnCYP18-4 | 58.35 | 542.31 | 3 | 1623.90 | 2.06 | HVVFGQVVEGLDVVK |  |
| BnCYP18-4 | 40.51 | 417.70 | 2 | 833.39 | 1.84 | TDWLDGK |  |
| BnCYP18-4 | 62.77 | 814.10 | 3 | 2439.28 | 1.87 | TDWLDGKHVVFGQVVEGLDVVK |  |
| BnCYP18-4 | 62.77 | 610.83 | 4 | 2439.28 | 2.01 | TDWLDGKHVVFGQVVEGLDVVK |  |
| BnCYP18-4 | 62.74 | 1220.65 | 2 | 2439.29 | 3.06 | TDWLDGKHVVFGQVVEGLDVVK |  |
| BnCYP18-4 | 43.36 | 485.26 | 3 | 1452.75 | -1.56 | IVMELYTDKTPK | [3] Oxidation (M) |
| BnCYP18-4 | 43.36 | 727.38 | 2 | 1452.75 | -1.55 | IVMELYTDKTPK | [3] Oxidation (M) |
| BnCYP18-4 | 33.54 | 727.38 | 2 | 1452.75 | -1.80 | IVMELYTDKTPK | [3] Oxidation (M) |
| BnCYP18-4 | 6.20 | 413.75 | 2 | 825.49 | -0.63 | GKPLHFK |  |
| BnCYP18-4 | 7.22 | 413.75 | 2 | 825.49 | -0.07 | GKPLHFK |  |
| BnCYP18-4 | 48.75 | 556.29 | 2 | 1110.57 | 3.03 | IVMELYTDK |  |
| BnCYP18-4 | 51.21 | 513.93 | 3 | 1538.76 | 3.39 | VFFDMTVDGKPAGR* |  |
| BnCYP18-4 | 51.21 | 770.39 | 2 | 1538.76 | 3.15 | VFFDMTVDGKPAGR* |  |
| BnCYP18-4 | 11.80 | 369.18 | 2 | 736.35 | 0.86 | TAENFR |  |
| BnCYP18-4 | 35.10 | 514.23 | 2 | 1026.44 | 1.98 | FADENFER* |  |
| BnCYP18-4 | 53.42 | 681.69 | 3 | 2042.05 | -0.74 | VGSASGKPSKPVVVADCGQLS* | [17] Carbamido-methyl (C) |
| **Accession** | **Retention time (min)** | **m/z** | **Charge** | **Measured mass (Da)** | **Mass error (ppm)** | **Sequence** | **Modifications** |
| BnCYP18-4 | 25.73 | 385.85 | 3 | 1154.54 | 0.32 | FADENFERK* |  |
| BnCYP18-4 | 25.70 | 578.28 | 2 | 1154.54 | 1.93 | FADENFERK* |  |
| BnCYP18-4 | 58.41 | 748.86 | 2 | 1495.70 | -8.49 | TAENFRALCTGEK | [9] Carbamido-methyl (C) |
| BnCYP18-4 | 58.38 | 499.57 | 3 | 1495.69 | -13.84 | TAENFRALCTGEK | [9] Carbamido-methyl (C) |
| BnCYP18-4 | 59.62 | 689.39 | 3 | 2065.14 | -6.67 | HVVFGQVVEGLDVVKAIEK |  |
| BnCYP18-4 | 45.02 | 627.87 | 2 | 1253.72 | -0.82 | GVGSKGKPLHFK* |  |
| BnCYP18-4 | 0.97 | 345.67 | 2 | 689.33 | 1.47 | GSSFHR |  |
| BnCYP18-4 | 0.43 | 345.67 | 2 | 689.33 | 3.57 | GSSFHR |  |
| BnCYP18-4 | 1.52 | 345.67 | 2 | 689.33 | 1.86 | GSSFHR |  |
| BnCYP18-4 | 7.02 | 389.69 | 2 | 777.37 | 0.37 | ALCTGEK | [3] Carbamido-methyl (C) |
| BnCYP18-5 | 35.75 | 403.71 | 2 | 805.40 | 2.47 | TSWLDGK* |  |
| BnCYP18-5 | 32.35 | 403.71 | 2 | 805.40 | 0.26 | TSWLDGK* |  |
| BnCYP18-5 | 51.99 | 587.78 | 2 | 1173.54 | 3.04 | VYFDMTVGDK* |  |
| BnCYP18-5 | 67.42 | 587.78 | 2 | 1173.54 | 3.60 | VYFDMTVGDK* |  |
| BnCYP18-5 | 58.12 | 719.38 | 3 | 2155.12 | 2.78 | HVVFGQVVEGMDVVRDIEK* |  |
| BnCYP18-5 | 58.09 | 1078.56 | 2 | 2155.11 | 1.50 | HVVFGQVVEGMDVVRDIEK* |  |
| BnCYP18-5 | 58.09 | 539.78 | 4 | 2155.11 | -1.75 | HVVFGQVVEGMDVVRDIEK* |  |
| BnCYP18-5 | 25.73 | 513.77 | 2 | 1025.52 | 2.53 | FKDENFVK |  |
| BnCYP18-5 | 25.70 | 342.85 | 3 | 1025.52 | 2.10 | FKDENFVK |  |
| BnCYP18-5 | 40.15 | 513.77 | 2 | 1025.52 | 1.44 | FKDENFVK |  |
| BnCYP18-5 | 61.21 | 1049.51 | 2 | 2097.01 | 2.05 | IVMELYADTVPETAENFR* |  |
| BnCYP18-5 | 61.21 | 700.01 | 3 | 2097.01 | 1.95 | IVMELYADTVPETAENFR* |  |
| BnCYP18-5 | 47.68 | 510.59 | 3 | 1528.74 | 0.79 | VYFDMTVGDKAAGR* |  |
| BnCYP18-5 | 47.68 | 765.38 | 2 | 1528.74 | 2.01 | VYFDMTVGDKAAGR* |  |
| BnCYP18-5 | 54.45 | 557.63 | 3 | 1669.87 | 3.11 | HVVFGQVVEGMDVVR* |  |
| BnCYP18-5 | 54.45 | 835.94 | 2 | 1669.86 | 2.30 | HVVFGQVVEGMDVVR* |  |
| BnCYP18-5 | 5.06 | 310.51 | 3 | 928.51 | -0.52 | SGKPLHYK |  |
| BnCYP18-5 | 5.33 | 310.51 | 3 | 928.51 | -0.77 | SGKPLHYK |  |
| BnCYP18-5 | 5.33 | 465.26 | 2 | 928.51 | -0.55 | SGKPLHYK |  |
| BnCYP18-5 | 5.06 | 465.26 | 2 | 928.51 | -0.24 | SGKPLHYK |  |
| BnCYP18-5 | 6.77 | 310.51 | 3 | 928.51 | -0.47 | SGKPLHYK |  |
| BnCYP18-5 | 4.63 | 310.51 | 3 | 928.51 | 0.36 | SGKPLHYK |  |
| BnCYP18-5 | 6.77 | 465.26 | 2 | 928.51 | 0.02 | SGKPLHYK |  |
| BnCYP18-5 | 0.07 | 310.51 | 3 | 928.51 | 0.95 | SGKPLHYK |  |
| BnCYP18-5 | 5.39 | 337.67 | 2 | 673.33 | -0.58 | GSAFHR |  |
| BnCYP18-5 | 4.81 | 337.67 | 2 | 673.33 | -0.62 | GSAFHR |  |
| BnCYP18-5 | 6.77 | 337.67 | 2 | 673.33 | -1.14 | GSAFHR |  |
| BnCYP18-5 | 5.76 | 337.67 | 2 | 673.33 | -1.11 | GSAFHR |  |
| BnCYP18-5 | 3.93 | 337.67 | 2 | 673.33 | 0.29 | GSAFHR |  |
| BnCYP18-5 | 3.05 | 337.67 | 2 | 673.33 | -0.06 | GSAFHR |  |
| BnCYP18-5 | 0.07 | 337.67 | 2 | 673.33 | 0.66 | GSAFHR |  |
| BnCYP18-5 | 54.60 | 567.30 | 2 | 1132.58 | -9.93 | ALCTGEKGIGK | [3] Carbamido-methyl (C) |
| BnCYP18-5 | 18.21 | 567.31 | 2 | 1132.60 | 8.39 | ALCTGEKGIGK | [3] Carbamido-methyl (C) |
| **Accession** | **Retention time (min)** | **m/z** | **Charge** | **Measured mass (Da)** | **Mass error (ppm)** | **Sequence** | **Modifications** |
| BnCYP18-5 | 60.16 | 820.09 | 3 | 2457.26 | 3.70 | TSWLDGKHVVFGQVVEGMDVVR* |  |
| BnCYP18-5 | 60.16 | 615.32 | 4 | 2457.25 | 2.90 | TSWLDGKHVVFGQVVEGMDVVR* |  |
| BnCYP18-5 | 52.99 | 724.71 | 3 | 2171.11 | 3.19 | HVVFGQVVEGMDVVRDIEK* | [11] Oxidation (M) |
| BnCYP18-5 | 57.28 | 1057.51 | 2 | 2113.01 | 2.84 | IVMELYADTVPETAENFR* | [3] Oxidation (M) |
| BnCYP18-5 | 57.30 | 705.34 | 3 | 2113.01 | 3.01 | IVMELYADTVPETAENFR* | [3] Oxidation (M) |
| BnCYP18-5 | 61.54 | 1057.51 | 2 | 2113.01 | 0.98 | IVMELYADTVPETAENFR* | [3] Oxidation (M) |
| BnCYP18-5 | 47.71 | 515.92 | 3 | 1544.73 | 2.15 | VYFDMTVGDKAAGR* | [5] Oxidation (M) |
| BnCYP18-5 | 47.66 | 773.37 | 2 | 1544.73 | 1.98 | VYFDMTVGDKAAGR* | [5] Oxidation (M) |
| BnCYP18-5 | 52.04 | 595.77 | 2 | 1189.54 | 2.32 | VYFDMTVGDK* | [5] Oxidation (M) |
| BnCYP18-5 | 40.91 | 595.77 | 2 | 1189.54 | 2.16 | VYFDMTVGDK* | [5] Oxidation (M) |
| BnCYP18-5 | 16.99 | 371.22 | 3 | 1110.63 | -0.13 | GSAFHRVIPK* |  |
| BnCYP18-5 | 17.28 | 556.32 | 2 | 1110.63 | -0.41 | GSAFHRVIPK* |  |
| BnCYP18-5 | 23.93 | 665.80 | 4 | 2659.17 | 5.87 | FMCQGGDFTAGNGTGGESIYGMKFK* | [3] Carbamido-methyl (C) |
| BnCYP18-5 | 57.46 | 947.11 | 3 | 2838.31 | 2.62 | HTGPGILSMANAGSNTNGSQFFICTEK* | [24] Carbamido-methyl (C) |
| BnCYP18-5 | 21.18 | 376.19 | 2 | 750.36 | 1.19 | DENFVK |  |
| BnCYP18-5 | 20.25 | 376.19 | 2 | 750.36 | 0.91 | DENFVK |  |
| BnCYP18-5 | 54.89 | 795.67 | 3 | 2383.99 | 2.36 | FMCQGGDFTAGNGTGGESIYGMK* | [3] Carbamido-methyl (C) |
| BnCYP18-5 | 12.31 | 440.23 | 2 | 878.45 | 0.42 | DENFVKK |  |
| BnCYP18-5 | 10.88 | 396.97 | 4 | 1583.83 | 0.43 | SGKPLHYKGSAFHR* |  |
| BnCYP18-5 | 10.81 | 528.95 | 3 | 1583.83 | 1.04 | SGKPLHYKGSAFHR* |  |
| BnCYP18-5 | 7.02 | 389.69 | 2 | 777.37 | 0.37 | ALCTGEK | [3] Carbamido-methyl (C) |
| BnCYP18-5 | 54.06 | 989.81 | 3 | 2966.41 | 1.70 | KHTGPGILSMANAGSNTNGSQFFICTEK* | [25] Carbamido-methyl (C) |
| BnCYP18-6 | 43.63 | 479.93 | 3 | 1436.76 | 2.49 | IVMELYTDKTPK |  |
| BnCYP18-6 | 43.63 | 719.39 | 2 | 1436.76 | 1.85 | IVMELYTDKTPK |  |
| BnCYP18-6 | 42.95 | 479.93 | 3 | 1436.77 | 5.57 | IVMELYTDKTPK |  |
| BnCYP18-6 | 56.23 | 542.31 | 3 | 1623.90 | 3.19 | HVVFGQVVEGLDVVK |  |
| BnCYP18-6 | 56.23 | 812.96 | 2 | 1623.90 | 2.25 | HVVFGQVVEGLDVVK |  |
| BnCYP18-6 | 58.35 | 542.31 | 3 | 1623.90 | 2.06 | HVVFGQVVEGLDVVK |  |
| BnCYP18-6 | 40.51 | 417.70 | 2 | 833.39 | 1.84 | TDWLDGK |  |
| BnCYP18-6 | 58.62 | 761.40 | 2 | 1520.78 | 2.59 | VFFDININGQAAGR* |  |
| BnCYP18-6 | 62.77 | 814.10 | 3 | 2439.28 | 1.87 | TDWLDGKHVVFGQVVEGLDVVK |  |
| BnCYP18-6 | 62.77 | 610.83 | 4 | 2439.28 | 2.01 | TDWLDGKHVVFGQVVEGLDVVK |  |
| BnCYP18-6 | 62.74 | 1220.65 | 2 | 2439.29 | 3.06 | TDWLDGKHVVFGQVVEGLDVVK |  |
| BnCYP18-6 | 43.36 | 485.26 | 3 | 1452.75 | -1.56 | IVMELYTDKTPK | [3] Oxidation (M) |
| BnCYP18-6 | 43.36 | 727.38 | 2 | 1452.75 | -1.55 | IVMELYTDKTPK | [3] Oxidation (M) |
| BnCYP18-6 | 33.54 | 727.38 | 2 | 1452.75 | -1.80 | IVMELYTDKTPK | [3] Oxidation (M) |
| BnCYP18-6 | 6.20 | 413.75 | 2 | 825.49 | -0.63 | GKPLHFK |  |
| BnCYP18-6 | 7.22 | 413.75 | 2 | 825.49 | -0.07 | GKPLHFK |  |
| BnCYP18-6 | 48.75 | 556.29 | 2 | 1110.57 | 3.03 | IVMELYTDK |  |
| BnCYP18-6 | 11.80 | 369.18 | 2 | 736.35 | 0.86 | TAENFR |  |
| BnCYP18-6 | 5.33 | 318.87 | 3 | 953.58 | -0.32 | KGKPLHFK |  |
| BnCYP18-6 | 5.33 | 477.80 | 2 | 953.58 | -0.01 | KGKPLHFK |  |
| **Accession** | **Retention time (min)** | **m/z** | **Charge** | **Measured mass (Da)** | **Mass error (ppm)** | **Sequence** | **Modifications** |
| BnCYP18-6 | 4.70 | 477.80 | 2 | 953.58 | 1.40 | KGKPLHFK |  |
| BnCYP18-6 | 33.97 | 607.28 | 2 | 1212.55 | 5.40 | FEDENFERK |  |
| BnCYP18-6 | 33.92 | 405.19 | 3 | 1212.54 | 3.17 | FEDENFERK |  |
| BnCYP18-6 | 25.88 | 405.19 | 3 | 1212.54 | 0.75 | FEDENFERK |  |
| BnCYP18-6 | 25.86 | 607.28 | 2 | 1212.54 | 0.00 | FEDENFERK |  |
| BnCYP18-6 | 58.41 | 748.86 | 2 | 1495.70 | -8.49 | TAENFRALCTGEK | [9] Carbamido-methyl (C) |
| BnCYP18-6 | 58.38 | 499.57 | 3 | 1495.69 | -13.84 | TAENFRALCTGEK | [9] Carbamido-methyl (C) |
| BnCYP18-6 | 59.62 | 689.39 | 3 | 2065.14 | -6.67 | HVVFGQVVEGLDVVKAIEK |  |
| BnCYP18-6 | 64.22 | 1283.22 | 3 | 3846.64 | -1.54 | VIPSFMCQGGDFTAGNGTGGESIYGDKFEDENFER* | [6] Oxidation (M) [7] Carbamido-methyl (C) |
| BnCYP18-6 | 0.97 | 345.67 | 2 | 689.33 | 1.47 | GSSFHR |  |
| BnCYP18-6 | 0.43 | 345.67 | 2 | 689.33 | 3.57 | GSSFHR |  |
| BnCYP18-6 | 1.52 | 345.67 | 2 | 689.33 | 1.86 | GSSFHR |  |
| BnCYP18-6 | 37.13 | 691.69 | 3 | 2072.06 | 1.85 | VGSSSGKPSKPVVIADCGQLS | [17] Carbamido-methyl (C) |
| BnCYP18-6 | 54.62 | 932.11 | 3 | 2793.30 | 1.49 | HTGPGNLSMANAGANTNGSQFFICTVK | [24] Carbamido-methyl (C) |
| BnCYP18-6 | 7.02 | 389.69 | 2 | 777.37 | 0.37 | ALCTGEK | [3] Carbamido-methyl (C) |
| BnCYP18-7 | 43.63 | 479.93 | 3 | 1436.76 | 2.49 | IVMELYTDKTPK |  |
| BnCYP18-7 | 43.63 | 719.39 | 2 | 1436.76 | 1.85 | IVMELYTDKTPK |  |
| BnCYP18-7 | 42.95 | 479.93 | 3 | 1436.77 | 5.57 | IVMELYTDKTPK |  |
| BnCYP18-7 | 56.23 | 542.31 | 3 | 1623.90 | 3.19 | HVVFGQVVEGLDVVK |  |
| BnCYP18-7 | 56.23 | 812.96 | 2 | 1623.90 | 2.25 | HVVFGQVVEGLDVVK |  |
| BnCYP18-7 | 58.35 | 542.31 | 3 | 1623.90 | 2.06 | HVVFGQVVEGLDVVK |  |
| BnCYP18-7 | 40.51 | 417.70 | 2 | 833.39 | 1.84 | TDWLDGK |  |
| BnCYP18-7 | 58.89 | 747.89 | 2 | 1493.77 | 2.99 | VFFDISINGQAAGR* |  |
| BnCYP18-7 | 63.65 | 747.89 | 2 | 1493.77 | 4.15 | VFFDISINGQAAGR* |  |
| BnCYP18-7 | 62.77 | 814.10 | 3 | 2439.28 | 1.87 | TDWLDGKHVVFGQVVEGLDVVK |  |
| BnCYP18-7 | 62.77 | 610.83 | 4 | 2439.28 | 2.01 | TDWLDGKHVVFGQVVEGLDVVK |  |
| BnCYP18-7 | 62.74 | 1220.65 | 2 | 2439.29 | 3.06 | TDWLDGKHVVFGQVVEGLDVVK |  |
| BnCYP18-7 | 43.36 | 485.26 | 3 | 1452.75 | -1.56 | IVMELYTDKTPK | [3] Oxidation (M) |
| BnCYP18-7 | 43.36 | 727.38 | 2 | 1452.75 | -1.55 | IVMELYTDKTPK | [3] Oxidation (M) |
| BnCYP18-7 | 33.54 | 727.38 | 2 | 1452.75 | -1.80 | IVMELYTDKTPK | [3] Oxidation (M) |
| BnCYP18-7 | 6.20 | 413.75 | 2 | 825.49 | -0.63 | GKPLHFK |  |
| BnCYP18-7 | 7.22 | 413.75 | 2 | 825.49 | -0.07 | GKPLHFK |  |
| BnCYP18-7 | 48.75 | 556.29 | 2 | 1110.57 | 3.03 | IVMELYTDK |  |
| BnCYP18-7 | 11.80 | 369.18 | 2 | 736.35 | 0.86 | TAENFR |  |
| BnCYP18-7 | 5.33 | 318.87 | 3 | 953.58 | -0.32 | KGKPLHFK |  |
| BnCYP18-7 | 5.33 | 477.80 | 2 | 953.58 | -0.01 | KGKPLHFK |  |
| BnCYP18-7 | 4.70 | 477.80 | 2 | 953.58 | 1.40 | KGKPLHFK |  |
| BnCYP18-7 | 33.97 | 607.28 | 2 | 1212.55 | 5.40 | FEDENFERK |  |
| BnCYP18-7 | 33.92 | 405.19 | 3 | 1212.54 | 3.17 | FEDENFERK |  |
| BnCYP18-7 | 25.88 | 405.19 | 3 | 1212.54 | 0.75 | FEDENFERK |  |
| BnCYP18-7 | 25.86 | 607.28 | 2 | 1212.54 | 0.00 | FEDENFERK |  |

| **Accession** | **Retention time (min)** | **m/z** | **Charge** | **Measured mass (Da)** | **Mass error (ppm)** | **Sequence** | **Modifications** |
| --- | --- | --- | --- | --- | --- | --- | --- |
| BnCYP18-7 | 58.41 | 748.86 | 2 | 1495.70 | -8.49 | TAENFRALCTGEK | [9] Carbamido-methyl (C) |
| BnCYP18-7 | 58.38 | 499.57 | 3 | 1495.69 | -13.84 | TAENFRALCTGEK | [9] Carbamido-methyl (C) |
| BnCYP18-7 | 59.62 | 689.39 | 3 | 2065.14 | -6.67 | HVVFGQVVEGLDVVKAIEK |  |
| BnCYP18-7 | 0.97 | 345.67 | 2 | 689.33 | 1.47 | GSSFHR |  |
| BnCYP18-7 | 0.43 | 345.67 | 2 | 689.33 | 3.57 | GSSFHR |  |
| BnCYP18-7 | 1.52 | 345.67 | 2 | 689.33 | 1.86 | GSSFHR |  |
| BnCYP18-7 | 37.13 | 691.69 | 3 | 2072.06 | 1.85 | VGSSSGKPSKPVVIADCGQLS | [17] Carbamido-methyl (C) |
| BnCYP18-7 | 54.62 | 932.11 | 3 | 2793.30 | 1.49 | HTGPGNLSMANAGANTNGSQFFICTVK | [24] Carbamido-methyl (C) |
| BnCYP18-7 | 7.02 | 389.69 | 2 | 777.37 | 0.37 | ALCTGEK | [3] Carbamido-methyl (C) |
| BnCYP19-1 | 33.77 | 511.75 | 2 | 1021.49 | -0.93 | AMEKVGSER* | [2] Oxidation (M) |
| BnCYP19-1 | 10.45 | 343.70 | 2 | 685.39 | 0.50 | HVVFGK |  |
| BnCYP19-1 | 39.64 | 459.58 | 3 | 1375.71 | -3.49 | VVMELFADVTPR |  |
| BnCYP19-1 | 39.66 | 688.86 | 2 | 1375.71 | -3.67 | VVMELFADVTPR |  |
| BnCYP19-1 | 61.08 | 688.87 | 2 | 1375.73 | 6.78 | VVMELFADVTPR |  |
| BnCYP19-1 | 63.30 | 698.33 | 4 | 2789.29 | 0.35 | IIPGFMCQGGDFTRGNGTGGESIYGAK* | [7] Carbamido-methyl (C) |
| BnCYP19-1 | 63.30 | 930.77 | 3 | 2789.29 | -0.96 | IIPGFMCQGGDFTRGNGTGGESIYGAK* | [7] Carbamido-methyl (C) |
| BnCYP19-1 | 63.28 | 558.87 | 5 | 2789.29 | 0.04 | IIPGFMCQGGDFTRGNGTGGESIYGAK* | [7] Carbamido-methyl (C) |
| BnCYP19-1 | 5.39 | 337.67 | 2 | 673.33 | -0.58 | GSAFHR |  |
| BnCYP19-1 | 4.81 | 337.67 | 2 | 673.33 | -0.62 | GSAFHR |  |
| BnCYP19-1 | 6.77 | 337.67 | 2 | 673.33 | -1.14 | GSAFHR |  |
| BnCYP19-1 | 5.76 | 337.67 | 2 | 673.33 | -1.11 | GSAFHR |  |
| BnCYP19-1 | 3.93 | 337.67 | 2 | 673.33 | 0.29 | GSAFHR |  |
| BnCYP19-1 | 3.05 | 337.67 | 2 | 673.33 | -0.06 | GSAFHR |  |
| BnCYP19-1 | 0.07 | 337.67 | 2 | 673.33 | 0.66 | GSAFHR |  |
| BnCYP19-1 | 33.10 | 496.78 | 2 | 991.54 | 2.01 | VVDGYNVVK |  |
| BnCYP19-1 | 66.22 | 526.31 | 2 | 1050.61 | 1.47 | VFFDILIGK |  |
| BnCYP19-1 | 21.70 | 351.21 | 3 | 1050.62 | 4.92 | VFFDILIGK |  |
| BnCYP19-1 | 21.89 | 316.18 | 2 | 630.35 | 1.99 | ALHYK |  |
| BnCYP19-1 | 20.33 | 316.18 | 2 | 630.35 | 0.59 | ALHYK |  |
| BnCYP19-1 | 34.31 | 568.76 | 2 | 1135.50 | -0.02 | NETSEVSNKE* |  |
| BnCYP19-1 | 56.82 | 463.91 | 3 | 1388.70 | -9.31 | ALCTGEKGIGQAGK | [3] Carbamido-methyl (C) |
| BnCYP19-1 | 22.75 | 605.79 | 2 | 1209.57 | 2.86 | GNGTGGESIYGAK* |  |
| BnCYP19-1 | 7.02 | 389.69 | 2 | 777.37 | 0.37 | ALCTGEK | [3] Carbamido-methyl (C) |
| BnCYP19-2 | 10.45 | 343.70 | 2 | 685.39 | 0.50 | HVVFGK |  |
| BnCYP19-2 | 39.64 | 459.58 | 3 | 1375.71 | -3.49 | VVMELFADVTPR |  |
| BnCYP19-2 | 39.66 | 688.86 | 2 | 1375.71 | -3.67 | VVMELFADVTPR |  |
| BnCYP19-2 | 61.08 | 688.87 | 2 | 1375.73 | 6.78 | VVMELFADVTPR |  |
| BnCYP19-2 | 46.87 | 655.99 | 3 | 1964.96 | -0.21 | VVDGYNVVKAMENVGSER* |  |
| BnCYP19-2 | 46.89 | 983.49 | 2 | 1964.97 | 1.54 | VVDGYNVVKAMENVGSER* |  |
| BnCYP19-2 | 5.39 | 337.67 | 2 | 673.33 | -0.58 | GSAFHR |  |
| BnCYP19-2 | 4.81 | 337.67 | 2 | 673.33 | -0.62 | GSAFHR |  |
| BnCYP19-2 | 6.77 | 337.67 | 2 | 673.33 | -1.14 | GSAFHR |  |
| **Accession** | **Retention time (min)** | **m/z** | **Charge** | **Measured mass (Da)** | **Mass error (ppm)** | **Sequence** | **Modifications** |
| BnCYP19-2 | 5.76 | 337.67 | 2 | 673.33 | -1.11 | GSAFHR |  |
| BnCYP19-2 | 3.93 | 337.67 | 2 | 673.33 | 0.29 | GSAFHR |  |
| BnCYP19-2 | 3.05 | 337.67 | 2 | 673.33 | -0.06 | GSAFHR |  |
| BnCYP19-2 | 0.07 | 337.67 | 2 | 673.33 | 0.66 | GSAFHR |  |
| BnCYP19-2 | 33.10 | 496.78 | 2 | 991.54 | 2.01 | VVDGYNVVK |  |
| BnCYP19-2 | 66.22 | 526.31 | 2 | 1050.61 | 1.47 | VFFDILIGK |  |
| BnCYP19-2 | 21.70 | 351.21 | 3 | 1050.62 | 4.92 | VFFDILIGK |  |
| BnCYP19-2 | 21.89 | 316.18 | 2 | 630.35 | 1.99 | ALHYK |  |
| BnCYP19-2 | 20.33 | 316.18 | 2 | 630.35 | 0.59 | ALHYK |  |
| BnCYP19-2 | 56.82 | 463.91 | 3 | 1388.70 | -9.31 | ALCTGEKGIGQAGK | [3] Carbamido-methyl (C) |
| BnCYP19-2 | 7.02 | 389.69 | 2 | 777.37 | 0.37 | ALCTGEK | [3] Carbamido-methyl (C) |
| BnCYP21-1 | 40.38 | 496.78 | 2 | 991.55 | 0.90 | VTIHANPLAG* |  |
| BnCYP21-1 | 44.52 | 683.33 | 2 | 1364.64 | 1.66 | GFMIQGGDPTGTGK* |  |
| BnCYP21-1 | 30.41 | 495.60 | 3 | 1483.78 | 2.44 | TQTGAGDRPLAEIR* |  |
| BnCYP21-1 | 30.27 | 742.90 | 2 | 1483.78 | 1.88 | TQTGAGDRPLAEIR* |  |
| BnCYP21-1 | 59.62 | 510.61 | 3 | 1528.80 | 2.97 | VIHGFEVLDIMEK* |  |
| BnCYP21-1 | 55.90 | 496.61 | 3 | 1486.80 | 2.85 | QPHLNGLYTIFGK* |  |
| BnCYP21-1 | 30.11 | 403.22 | 2 | 804.42 | 5.68 | GGTSIWGK* |  |
| BnCYP21-3 | 10.45 | 343.70 | 2 | 685.39 | 0.50 | HVVFGK |  |
| BnCYP21-3 | 5.06 | 310.51 | 3 | 928.51 | -0.52 | SGKPLHYK |  |
| BnCYP21-3 | 5.33 | 310.51 | 3 | 928.51 | -0.77 | SGKPLHYK |  |
| BnCYP21-3 | 5.33 | 465.26 | 2 | 928.51 | -0.55 | SGKPLHYK |  |
| BnCYP21-3 | 5.06 | 465.26 | 2 | 928.51 | -0.24 | SGKPLHYK |  |
| BnCYP21-3 | 6.77 | 310.51 | 3 | 928.51 | -0.47 | SGKPLHYK |  |
| BnCYP21-3 | 4.63 | 310.51 | 3 | 928.51 | 0.36 | SGKPLHYK |  |
| BnCYP21-3 | 6.77 | 465.26 | 2 | 928.51 | 0.02 | SGKPLHYK |  |
| BnCYP21-3 | 0.07 | 310.51 | 3 | 928.51 | 0.95 | SGKPLHYK |  |
| BnCYP21-3 | 37.61 | 435.71 | 2 | 869.40 | 4.60 | FADENFK |  |
| BnCYP21-3 | 38.86 | 435.71 | 2 | 869.40 | 4.13 | FADENFK |  |
| BnCYP21-3 | 29.78 | 435.70 | 2 | 869.39 | 1.37 | FADENFK |  |
| BnCYP21-3 | 11.80 | 369.18 | 2 | 736.35 | 0.86 | TAENFR |  |
| BnCYP21-3 | 46.55 | 556.29 | 2 | 1110.57 | 1.91 | FADENFKLK |  |
| BnCYP21-3 | 5.68 | 556.30 | 2 | 1110.58 | 6.33 | FADENFKLK |  |
| BnCYP21-3 | 6.84 | 556.30 | 2 | 1110.58 | 6.04 | FADENFKLK |  |
| BnCYP21-3 | 19.09 | 371.55 | 3 | 1111.62 | 5.47 | VVIADSGELPL* |  |
| BnCYP21-3 | 19.27 | 556.82 | 2 | 1111.62 | 4.93 | VVIADSGELPL* |  |
| BnCYP21-3 | 50.50 | 416.73 | 2 | 831.45 | 6.34 | QSGTPKSK* |  |
| BnCYP21-3 | 33.97 | 415.55 | 3 | 1243.64 | 0.99 | IEAEGKQSGTPK* |  |
| BnCYP21-3 | 34.02 | 622.83 | 2 | 1243.64 | 1.09 | IEAEGKQSGTPK* |  |
| BnCYP21-3 | 42.07 | 569.30 | 2 | 1136.59 | 2.39 | VVQGMDVVYK* |  |
| BnCYP21-3 | 53.80 | 416.77 | 2 | 831.52 | 2.35 | VVIGLFGK* |  |
| BnCYP21-3 | 58.41 | 748.86 | 2 | 1495.70 | -8.49 | TAENFRALCTGEK | [9] Carbamido-methyl (C) |
| BnCYP21-3 | 58.38 | 499.57 | 3 | 1495.69 | -13.84 | TAENFRALCTGEK | [9] Carbamido-methyl (C) |
| **Accession** | **Retention time (min)** | **m/z** | **Charge** | **Measured mass (Da)** | **Mass error (ppm)** | **Sequence** | **Modifications** |
| BnCYP21-3 | 60.02 | 947.79 | 3 | 2840.33 | 2.95 | IIPSFMIQGGDFTHGNGMGGESIYGQK* |  |
| BnCYP21-3 | 60.35 | 711.08 | 4 | 2840.30 | -10.68 | IIPSFMIQGGDFTHGNGMGGESIYGQK* |  |
| BnCYP21-3 | 60.35 | 947.77 | 3 | 2840.30 | -9.80 | IIPSFMIQGGDFTHGNGMGGESIYGQK* |  |
| BnCYP21-3 | 7.02 | 389.69 | 2 | 777.37 | 0.37 | ALCTGEK | [3] Carbamido-methyl (C) |
| BnCYP21-4 | 10.45 | 343.70 | 2 | 685.39 | 0.50 | HVVFGK |  |
| BnCYP21-4 | 38.88 | 354.20 | 2 | 706.38 | 1.03 | MARSVK* | [1] Oxidation (M) |
| BnCYP21-4 | 38.44 | 354.20 | 2 | 706.38 | 1.36 | MARSVK* | [1] Oxidation (M) |
| BnCYP21-4 | 18.92 | 431.90 | 3 | 1292.69 | -2.97 | EAGRIVMGLFGK | [7] Oxidation (M) |
| BnCYP21-4 | 18.89 | 647.35 | 2 | 1292.69 | -3.55 | EAGRIVMGLFGK | [7] Oxidation (M) |
| BnCYP21-4 | 18.98 | 324.18 | 4 | 1292.69 | -3.51 | EAGRIVMGLFGK | [7] Oxidation (M) |
| BnCYP21-4 | 37.61 | 435.71 | 2 | 869.40 | 4.60 | FADENFK |  |
| BnCYP21-4 | 38.86 | 435.71 | 2 | 869.40 | 4.13 | FADENFK |  |
| BnCYP21-4 | 29.78 | 435.70 | 2 | 869.39 | 1.37 | FADENFK |  |
| BnCYP21-4 | 11.80 | 369.18 | 2 | 736.35 | 0.86 | TAENFR |  |
| BnCYP21-4 | 46.55 | 556.29 | 2 | 1110.57 | 1.91 | FADENFKLK |  |
| BnCYP21-4 | 5.68 | 556.30 | 2 | 1110.58 | 6.33 | FADENFKLK |  |
| BnCYP21-4 | 6.84 | 556.30 | 2 | 1110.58 | 6.04 | FADENFKLK |  |
| BnCYP21-4 | 44.20 | 380.89 | 3 | 1139.64 | -4.36 | VVIVDSGELPL |  |
| BnCYP21-4 | 44.91 | 380.89 | 3 | 1139.64 | -4.17 | VVIVDSGELPL |  |
| BnCYP21-4 | 44.20 | 570.83 | 2 | 1139.64 | -3.39 | VVIVDSGELPL |  |
| BnCYP21-4 | 44.91 | 570.83 | 2 | 1139.64 | -3.26 | VVIVDSGELPL |  |
| BnCYP21-4 | 64.29 | 570.83 | 2 | 1139.65 | 2.17 | VVIVDSGELPL |  |
| BnCYP21-4 | 58.41 | 748.86 | 2 | 1495.70 | -8.49 | TAENFRALCTGEK | [9] Carbamido-methyl (C) |
| BnCYP21-4 | 58.38 | 499.57 | 3 | 1495.69 | -13.84 | TAENFRALCTGEK | [9] Carbamido-methyl (C) |
| BnCYP21-4 | 54.11 | 432.76 | 2 | 863.50 | 2.55 | IVMGLFGK |  |
| BnCYP21-4 | 21.89 | 316.18 | 2 | 630.35 | 1.99 | ALHYK |  |
| BnCYP21-4 | 20.33 | 316.18 | 2 | 630.35 | 0.59 | ALHYK |  |
| BnCYP21-4 | 44.68 | 555.80 | 2 | 1109.58 | 2.24 | VVTGMDVVYK* |  |
| BnCYP21-4 | 0.97 | 345.67 | 2 | 689.33 | 1.47 | GSSFHR |  |
| BnCYP21-4 | 0.43 | 345.67 | 2 | 689.33 | 3.57 | GSSFHR |  |
| BnCYP21-4 | 1.52 | 345.67 | 2 | 689.33 | 1.86 | GSSFHR |  |
| BnCYP21-4 | 7.02 | 389.69 | 2 | 777.37 | 0.37 | ALCTGEK | [3] Carbamido-methyl (C) |
| BnCYP21-9 | 10.45 | 343.70 | 2 | 685.39 | 0.50 | HVVFGK |  |
| BnCYP21-9 | 18.92 | 431.90 | 3 | 1292.69 | -2.97 | EAGRIVMGLFGK | [7] Oxidation (M) |
| BnCYP21-9 | 18.89 | 647.35 | 2 | 1292.69 | -3.55 | EAGRIVMGLFGK | [7] Oxidation (M) |
| BnCYP21-9 | 18.98 | 324.18 | 4 | 1292.69 | -3.51 | EAGRIVMGLFGK | [7] Oxidation (M) |
| BnCYP21-9 | 54.60 | 567.30 | 2 | 1132.58 | -9.93 | ALCTGEKGIGK | [3] Carbamido-methyl (C) |
| BnCYP21-9 | 18.21 | 567.31 | 2 | 1132.60 | 8.39 | ALCTGEKGIGK | [3] Carbamido-methyl (C) |
| BnCYP21-9 | 11.80 | 369.18 | 2 | 736.35 | 0.86 | TAENFR |  |
| BnCYP21-9 | 44.20 | 380.89 | 3 | 1139.64 | -4.36 | VVIVDSGELPL |  |
| BnCYP21-9 | 44.91 | 380.89 | 3 | 1139.64 | -4.17 | VVIVDSGELPL |  |
| BnCYP21-9 | 44.20 | 570.83 | 2 | 1139.64 | -3.39 | VVIVDSGELPL |  |
| **Accession** | **Retention time (min)** | **m/z** | **Charge** | **Measured mass (Da)** | **Mass error (ppm)** | **Sequence** | **Modifications** |
| BnCYP21-9 | 44.91 | 570.83 | 2 | 1139.64 | -3.26 | VVIVDSGELPL |  |
| BnCYP21-9 | 64.29 | 570.83 | 2 | 1139.65 | 2.17 | VVIVDSGELPL |  |
| BnCYP21-9 | 58.41 | 748.86 | 2 | 1495.70 | -8.49 | TAENFRALCTGEK | [9] Carbamido-methyl (C) |
| BnCYP21-9 | 58.38 | 499.57 | 3 | 1495.69 | -13.84 | TAENFRALCTGEK | [9] Carbamido-methyl (C) |
| BnCYP21-9 | 42.51 | 548.79 | 2 | 1095.57 | 2.91 | VVSGMDVVYK* |  |
| BnCYP21-9 | 35.15 | 366.20 | 3 | 1095.57 | 8.58 | VVSGMDVVYK* |  |
| BnCYP21-9 | 54.11 | 432.76 | 2 | 863.50 | 2.55 | IVMGLFGK |  |
| BnCYP21-9 | 21.89 | 316.18 | 2 | 630.35 | 1.99 | ALHYK |  |
| BnCYP21-9 | 20.33 | 316.18 | 2 | 630.35 | 0.59 | ALHYK |  |
| BnCYP21-9 | 0.97 | 345.67 | 2 | 689.33 | 1.47 | GSSFHR |  |
| BnCYP21-9 | 0.43 | 345.67 | 2 | 689.33 | 3.57 | GSSFHR |  |
| BnCYP21-9 | 1.52 | 345.67 | 2 | 689.33 | 1.86 | GSSFHR |  |
| BnCYP21-9 | 7.02 | 389.69 | 2 | 777.37 | 0.37 | ALCTGEK | [3] Carbamido-methyl (C) |
| BnCYP21-9 | 24.54 | 410.87 | 3 | 1229.58 | -6.14 | IEAEGNQSGTPK* |  |
| BnCYP22-2 | 11.80 | 369.18 | 2 | 736.35 | 0.86 | TAENFR |  |
| BnCYP22-2 | 57.28 | 567.80 | 2 | 1133.58 | 2.72 | MELFADIAPK |  |
| BnCYP22-2 | 61.10 | 650.82 | 2 | 1299.62 | 0.67 | DFMIQSGDFLK |  |
| BnCYP22-2 | 21.61 | 434.21 | 3 | 1299.61 | -5.88 | DFMIQSGDFLK |  |
| BnCYP22-2 | 21.61 | 650.81 | 2 | 1299.61 | -6.64 | DFMIQSGDFLK |  |
| BnCYP22-2 | 57.00 | 536.81 | 2 | 1071.61 | 0.95 | VLGDGLLVMR* |  |
| BnCYP22-2 | 24.28 | 469.93 | 3 | 1406.77 | 2.13 | IENVAVGPNNRPK |  |
| BnCYP22-2 | 24.28 | 704.39 | 2 | 1406.77 | 1.99 | IENVAVGPNNRPK |  |
| BnCYP22-2 | 33.64 | 569.79 | 2 | 1137.57 | 7.62 | QFCTGELRK | [3] Carbamido-methyl (C) |
| BnCYP22-2 | 28.10 | 505.74 | 2 | 1009.47 | 4.47 | QFCTGELR | [3] Carbamido-methyl (C) |
| BnCYP22-2 | 31.65 | 512.62 | 3 | 1534.85 | -4.75 | KIENVAVGPNNRPK |  |
| BnCYP22-2 | 31.65 | 768.43 | 2 | 1534.85 | -4.91 | KIENVAVGPNNRPK |  |
| BnCYP22-2 | 36.82 | 543.74 | 2 | 1085.47 | 3.14 | FDDENFTAK |  |
| BnCYP22-2 | 13.80 | 357.71 | 2 | 713.40 | 0.17 | HVVFGR |  |
| BnCYP22-2 | 6.43 | 608.82 | 2 | 1215.62 | 1.90 | ECQFHRVIK | [2] Carbamido-methyl (C) |
| BnCYP27-2 | 35.73 | 488.75 | 2 | 975.49 | 0.58 | NQRTACVK | [6] Carbamido-methyl (C) |
| BnCYP27-2 | 48.32 | 571.78 | 2 | 1141.54 | 6.96 | FEDENFTLK |  |
| BnCYP27-2 | 62.12 | 637.86 | 2 | 1273.71 | 2.43 | IVMGLFGDVVPK |  |
| BnCYP27-2 | 50.66 | 642.34 | 3 | 1924.00 | 2.30 | VTNKVYFDVEIGGEVAGK* |  |
| BnCYP27-2 | 20.96 | 680.35 | 2 | 1358.69 | -5.44 | HVVFGQVIEGMK | [11] Oxidation (M) |
| BnCYP27-2 | 29.49 | 338.69 | 2 | 675.36 | 2.13 | AFDVPK |  |
| BnCYP27-2 | 28.07 | 338.69 | 2 | 675.36 | 1.50 | AFDVPK |  |
| BnCYP27-2 | 13.12 | 338.69 | 2 | 675.36 | 5.69 | AFDVPK |  |
| BnCYP27-2 | 49.28 | 672.36 | 2 | 1342.71 | 2.23 | HVVFGQVIEGMK |  |
| BnCYP27-2 | 35.60 | 437.23 | 2 | 872.44 | 2.00 | TPWLDNK* |  |
| BnCYP27-2 | 18.45 | 383.20 | 2 | 764.38 | 2.11 | TVENFR |  |
| BnCYP27-2 | 0.97 | 345.67 | 2 | 689.33 | 1.47 | GSSFHR |  |
| BnCYP27-2 | 0.43 | 345.67 | 2 | 689.33 | 3.57 | GSSFHR |  |

| **Accession** | **Retention time (min)** | **m/z** | **Charge** | **Measured mass (Da)** | **Mass error (ppm)** | **Sequence** | **Modifications** |
| --- | --- | --- | --- | --- | --- | --- | --- |
| BnCYP27-2 | 1.52 | 345.67 | 2 | 689.33 | 1.86 | GSSFHR |  |
| BnCYP27-2 | 17.85 | 402.73 | 2 | 803.45 | 0.34 | AFDVPKK |  |
| BnCYP27-2 | 63.87 | 1218.07 | 2 | 2434.12 | 3.38 | DFMIQGGDFTEGNGTGGISIYGAK |  |
| BnCYP27-2 | 63.71 | 975.97 | 2 | 1949.92 | -7.80 | MASLSSMQMVHTSQIGVK | [9] Oxidation (M) |
| BnCYP27-3 | 25.22 | 504.26 | 4 | 2013.00 | 8.64 | MAAFSMTLSNPKAFSAAPR* | [1] Oxidation (M) |
| BnCYP27-3 | 47.07 | 567.64 | 3 | 1699.89 | 9.18 | HVVFGQVIEGMDVVR* | [11] Oxidation (M) |
| BnCYP27-3 | 17.42 | 404.73 | 2 | 807.45 | -0.19 | STVNFIK* |  |
| BnCYP27-3 | 28.07 | 545.27 | 3 | 1632.79 | 0.05 | KVVIADCGQLPMSEA* | [7] Carbamido-methyl (C)  [12] Oxidation (M) |
| BnCYP27-3 | 28.07 | 817.40 | 2 | 1632.78 | -3.81 | KVVIADCGQLPMSEA* | [7] Carbamido-methyl (C)  [12] Oxidation (M) |
| BnCYP27-3 | 24.65 | 628.32 | 3 | 1881.94 | 2.02 | AAFSMTLSNPKAFSAAPR* | [5] Oxidation (M) |
| BnCYP27-3 | 19.48 | 571.96 | 3 | 1712.84 | -0.18 | LIEEQETDRGDRPR* |  |
| BnCYP27-3 | 7.02 | 389.69 | 2 | 777.37 | 0.37 | ALCTGEK | [3] Carbamido-methyl (C) |
| BnCYP27-4 | 35.73 | 488.75 | 2 | 975.49 | 0.58 | NQRTACVK | [6] Carbamido-methyl (C) |
| BnCYP27-4 | 50.78 | 660.84 | 2 | 1319.67 | -4.11 | IEMGLFGDVVPK* | [3] Oxidation (M) |
| BnCYP27-4 | 48.32 | 571.78 | 2 | 1141.54 | 6.96 | FEDENFTLK |  |
| BnCYP27-4 | 55.59 | 689.70 | 3 | 2066.06 | 6.91 | IEMGLFGDVVPKTVENFR* | [3] Oxidation (M) |
| BnCYP27-4 | 55.62 | 1034.04 | 2 | 2066.07 | 7.00 | IEMGLFGDVVPKTVENFR* | [3] Oxidation (M) |
| BnCYP27-4 | 35.96 | 513.81 | 2 | 1025.60 | 2.02 | HVVFGQVIK* |  |
| BnCYP27-4 | 9.16 | 342.87 | 3 | 1025.60 | -4.26 | HVVFGQVIK* |  |
| BnCYP27-4 | 9.19 | 513.81 | 2 | 1025.60 | -3.13 | HVVFGQVIK* |  |
| BnCYP27-4 | 37.56 | 408.72 | 2 | 815.42 | 1.69 | TPWLDGK* |  |
| BnCYP27-4 | 29.49 | 338.69 | 2 | 675.36 | 2.13 | AFDVPK |  |
| BnCYP27-4 | 28.07 | 338.69 | 2 | 675.36 | 1.50 | AFDVPK |  |
| BnCYP27-4 | 13.12 | 338.69 | 2 | 675.36 | 5.69 | AFDVPK |  |
| BnCYP27-4 | 21.59 | 408.56 | 3 | 1222.67 | -0.30 | KQFPGAYATIK* |  |
| BnCYP27-4 | 21.64 | 306.67 | 4 | 1222.67 | -1.16 | KQFPGAYATIK* |  |
| BnCYP27-4 | 18.45 | 383.20 | 2 | 764.38 | 2.11 | TVENFR |  |
| BnCYP27-4 | 0.97 | 345.67 | 2 | 689.33 | 1.47 | GSSFHR |  |
| BnCYP27-4 | 0.43 | 345.67 | 2 | 689.33 | 3.57 | GSSFHR |  |
| BnCYP27-4 | 1.52 | 345.67 | 2 | 689.33 | 1.86 | GSSFHR |  |
| BnCYP27-4 | 23.15 | 548.29 | 2 | 1094.58 | -0.81 | QFPGAYATIK* |  |
| BnCYP27-4 | 22.78 | 548.30 | 2 | 1094.58 | -0.31 | QFPGAYATIK* |  |
| BnCYP27-4 | 17.85 | 402.73 | 2 | 803.45 | 0.34 | AFDVPKK |  |
| BnCYP27-4 | 63.87 | 1218.07 | 2 | 2434.12 | 3.38 | DFMIQGGDFTEGNGTGGISIYGAK |  |
| BnCYP27-4 | 63.71 | 975.97 | 2 | 1949.92 | -7.80 | MASLSSMQMVHTSQIGVK | [9] Oxidation (M) |
| BnCYP28-1 | 48.32 | 571.78 | 2 | 1141.54 | 6.96 | FEDENFTLK |  |
| BnCYP28-1 | 25.11 | 385.22 | 3 | 1152.63 | 7.12 | LNYATSFPLK* |  |
| BnCYP28-1 | 25.11 | 577.32 | 2 | 1152.63 | 7.68 | LNYATSFPLK* |  |
| BnCYP28-1 | 20.96 | 680.35 | 2 | 1358.69 | -5.44 | HVVFGQVIEGMK | [11] Oxidation (M) |
| BnCYP28-1 | 29.96 | 464.21 | 3 | 1389.62 | -6.42 | ASSSPSQMVHTSR | [8] Oxidation (M) |
| BnCYP28-1 | 29.93 | 695.82 | 2 | 1389.62 | -6.14 | ASSSPSQMVHTSR | [8] Oxidation (M) |

| **Accession** | **Retention time (min)** | **m/z** | **Charge** | **Measured mass (Da)** | **Mass error (ppm)** | **Sequence** | **Modifications** |
| --- | --- | --- | --- | --- | --- | --- | --- |
| BnCYP28-1 | 30.90 | 453.74 | 2 | 905.46 | -6.32 | ALCTGEKK | [3] Carbamido-methyl (C) |
| BnCYP28-1 | 49.28 | 672.36 | 2 | 1342.71 | 2.23 | HVVFGQVIEGMK |  |
| BnCYP28-1 | 18.45 | 383.20 | 2 | 764.38 | 2.11 | TVENFR |  |
| BnCYP28-1 | 0.97 | 345.67 | 2 | 689.33 | 1.47 | GSSFHR |  |
| BnCYP28-1 | 0.43 | 345.67 | 2 | 689.33 | 3.57 | GSSFHR |  |
| BnCYP28-1 | 1.52 | 345.67 | 2 | 689.33 | 1.86 | GSSFHR |  |
| BnCYP28-1 | 63.07 | 651.87 | 2 | 1301.72 | 2.49 | IVMGLFGDVVPR* |  |
| BnCYP28-1 | 18.76 | 507.25 | 3 | 1518.74 | 3.50 | LESQETRAMDVPK | [9] Oxidation (M) |
| BnCYP28-1 | 12.02 | 357.20 | 2 | 712.39 | 6.57 | TISGAHK* |  |
| BnCYP28-1 | 63.87 | 1218.07 | 2 | 2434.12 | 3.38 | DFMIQGGDFTEGNGTGGISIYGAK |  |
| BnCYP28-1 | 7.02 | 389.69 | 2 | 777.37 | 0.37 | ALCTGEK | [3] Carbamido-methyl (C) |
| BnCYP28-2 | 48.32 | 571.78 | 2 | 1141.54 | 6.96 | FEDENFTLK |  |
| BnCYP28-2 | 62.12 | 637.86 | 2 | 1273.71 | 2.43 | IVMGLFGDVVPK |  |
| BnCYP28-2 | 20.96 | 680.35 | 2 | 1358.69 | -5.44 | HVVFGQVIEGMK | [11] Oxidation (M) |
| BnCYP28-2 | 29.96 | 464.21 | 3 | 1389.62 | -6.42 | ASSSPSQMVHTSR | [8] Oxidation (M) |
| BnCYP28-2 | 29.93 | 695.82 | 2 | 1389.62 | -6.14 | ASSSPSQMVHTSR | [8] Oxidation (M) |
| BnCYP28-2 | 30.90 | 453.74 | 2 | 905.46 | -6.32 | ALCTGEKK | [3] Carbamido-methyl (C) |
| BnCYP28-2 | 49.28 | 672.36 | 2 | 1342.71 | 2.23 | HVVFGQVIEGMK |  |
| BnCYP28-2 | 18.45 | 383.20 | 2 | 764.38 | 2.11 | TVENFR |  |
| BnCYP28-2 | 0.97 | 345.67 | 2 | 689.33 | 1.47 | GSSFHR |  |
| BnCYP28-2 | 0.43 | 345.67 | 2 | 689.33 | 3.57 | GSSFHR |  |
| BnCYP28-2 | 1.52 | 345.67 | 2 | 689.33 | 1.86 | GSSFHR |  |
| BnCYP28-2 | 18.76 | 507.25 | 3 | 1518.74 | 3.50 | LESQETRAMDVPK | [9] Oxidation (M) |
| BnCYP28-2 | 14.96 | 509.77 | 2 | 1017.52 | 3.13 | RLESQETR* |  |
| BnCYP28-2 | 63.87 | 1218.07 | 2 | 2434.12 | 3.38 | DFMIQGGDFTEGNGTGGISIYGAK |  |
| BnCYP28-2 | 7.02 | 389.69 | 2 | 777.37 | 0.37 | ALCTGEK | [3] Carbamido-methyl (C) |
| BnCYP86-2 | 45.85 | 443.73 | 2 | 885.44 | -4.24 | EGPDLLDK* |  |
| BnCYP86-2 | 5.06 | 310.51 | 3 | 928.51 | -0.52 | SGKPLHYK |  |
| BnCYP86-2 | 5.33 | 310.51 | 3 | 928.51 | -0.77 | SGKPLHYK |  |
| BnCYP86-2 | 5.33 | 465.26 | 2 | 928.51 | -0.55 | SGKPLHYK |  |
| BnCYP86-2 | 5.06 | 465.26 | 2 | 928.51 | -0.24 | SGKPLHYK |  |
| BnCYP86-2 | 6.77 | 310.51 | 3 | 928.51 | -0.47 | SGKPLHYK |  |
| BnCYP86-2 | 4.63 | 310.51 | 3 | 928.51 | 0.36 | SGKPLHYK |  |
| BnCYP86-2 | 6.77 | 465.26 | 2 | 928.51 | 0.02 | SGKPLHYK |  |
| BnCYP86-2 | 0.07 | 310.51 | 3 | 928.51 | 0.95 | SGKPLHYK |  |
| BnCYP86-2 | 36.12 | 507.29 | 2 | 1012.57 | -8.87 | SASLGRGPLR* |  |
| BnCYP86-2 | 25.30 | 338.53 | 3 | 1012.58 | 4.88 | SASLGRGPLR* |  |
| BnCYP86-2 | 25.30 | 507.30 | 2 | 1012.58 | 5.46 | SASLGRGPLR* |  |
| BnCYP86-2 | 33.72 | 430.24 | 2 | 858.46 | 7.68 | NVIDDRK* |  |
| BnCYP86-2 | 18.17 | 453.26 | 2 | 904.50 | -5.59 | SLSRSISR* |  |
| BnCYP86-2 | 18.14 | 302.51 | 3 | 904.50 | -5.89 | SLSRSISR* |  |
| BnCYP86-2 | 30.58 | 507.77 | 2 | 1013.53 | -4.34 | KEGPDLLDK* |  |

| **Accession** | **Retention time (min)** | **m/z** | **Charge** | **Measured mass (Da)** | **Mass error (ppm)** | **Sequence** | **Modifications** |
| --- | --- | --- | --- | --- | --- | --- | --- |
| BnCYP86-2 | 33.28 | 531.77 | 2 | 1061.53 | 0.17 | SPESSRLMR* |  |
| BnCYP86-2 | 18.98 | 466.25 | 2 | 930.49 | -1.25 | SPLMSPKR* | [4] Oxidation (M) |
| BnCYP86-2 | 48.47 | 571.75 | 2 | 1141.49 | 6.25 | SSSSSSGNSSAGK* |  |
| BnCYP86-2 | 48.47 | 381.50 | 3 | 1141.49 | 5.14 | SSSSSSGNSSAGK* |  |
| BnCYP86-2 | 38.22 | 514.58 | 3 | 1540.70 | 4.38 | SPSKSSSSSSGNSSAGK* |  |
| BnCYP86-2 | 38.30 | 771.36 | 2 | 1540.70 | 4.29 | SPSKSSSSSSGNSSAGK* |  |
| BnCYP86-2 | 8.81 | 396.24 | 2 | 790.47 | 0.80 | KNVVFGK* |  |
| BnCYP86-2 | 19.62 | 396.24 | 2 | 790.47 | 1.45 | KNVVFGK* |  |
| BnCYP86-2 | 16.48 | 383.20 | 2 | 764.39 | -3.60 | SRSPYR* |  |
| BnCYP86-2 | 5.90 | 458.26 | 2 | 914.51 | 8.58 | SPLMSPKR* |  |
| BnCYP86-2 | 19.12 | 480.77 | 2 | 959.52 | 5.49 | SWSLSPKR* |  |
| BnCYP86-2 | 28.61 | 379.71 | 2 | 757.41 | 2.01 | SPSKSPR* |  |
| BnCYP86-2 | 30.34 | 609.83 | 2 | 1217.65 | 1.85 | SISRSPLMSPK* | [8] Oxidation (M) |
| BnCYP86-2 | 7.02 | 389.69 | 2 | 777.37 | 0.37 | ALCTGEK | [3] Carbamido-methyl (C) |
| BnCYP86-2 | 21.21 | 373.87 | 3 | 1118.60 | -8.65 | SVSRSPVLMK* | [9] Oxidation (M) |
